# Integration of transcriptomics and machine learning for insights into breast cancer: exploring lipid metabolism and immune interactions

**DOI:** 10.3389/fimmu.2024.1470167

**Published:** 2024-10-25

**Authors:** Xiaohan Chen, Jinfeng Yi, Lili Xie, Tong Liu, Baogang Liu, Meisi Yan

**Affiliations:** ^1^ Harbin Medical University Cancer Hospital, Harbin Medical University, Harbin, China; ^2^ Department of Basic Medical Sciences, Harbin Medical University, Harbin, China; ^3^ National Health Commission (NHC) Key Laboratory of Cell Transplantation, The First Affiliated Hospital of Harbin Medical University, Harbin, China

**Keywords:** breast cancer, tumor immune microenvironment, fatty acid metabolism, machine learning, omics, immune infiltration analysis

## Abstract

**Background:**

Breast cancer (BRCA) represents a substantial global health challenge marked by inadequate early detection rates. The complex interplay between the tumor immune microenvironment and fatty acid metabolism in BRCA requires further investigation to elucidate the specific role of lipid metabolism in this disease.

**Methods:**

We systematically integrated nine machine learning algorithms into 184 unique combinations to develop a consensus model for lipid metabolism-related prognostic genes (LMPGS). Additionally, transcriptomics analysis provided a comprehensive understanding of this prognostic signature. Using the ESTIMATE method, we evaluated immune infiltration among different risk subgroups and assessed their responsiveness to immunotherapy. Tailored treatments were screened for specific risk subgroups. Finally, we verified the expression of key genes through *in vitro* experiments.

**Results:**

We identified 259 differentially expressed genes (DEGs) related to lipid metabolism through analysis of the cancer genome atlas program (TCGA) database. Subsequently, via univariate Cox regression analysis and C-index analysis, we developed an optimal machine learning algorithm to construct a 21-gene LMPGS model. We used optimal cutoff values to divide the lipid metabolism prognostic gene scores into two groups according to high and low scores. Our study revealed distinct biological functions and mutation landscapes between high-scoring and low-scoring patients. The low-scoring group presented a greater immune score, whereas the high-scoring group presented enhanced responses to both immunotherapy and chemotherapy drugs. Single-cell analysis highlighted significant upregulation of CPNE3 in epithelial cells. Moreover, by employing molecular docking, we identified niclosamide as a potential targeted therapeutic drug. Finally, our experiments demonstrated high expression of MTMR9 and CPNE3 in BRCA and their significant correlation with prognosis.

**Conclusion:**

By employing bioinformatics and diverse machine learning algorithms, we successfully identified genes associated with lipid metabolism in BRCA and uncovered potential therapeutic agents, thereby offering novel insights into the mechanisms and treatment strategies for BRCA.

## 1 Introduction

BRCA is the most prevalent cancer and a primary cause of cancer-related death among women worldwide (1).It is widely acknowledged that this disease is heterogeneous at both the clinical and molecular levels (2, 3). Immunotherapy has emerged as the primary treatment (4) for many patients with BRCA and PD-1-or PD-L1- targeted drugs have shown some initial promise; however, when used as a single drug or in combination with traditional cytotoxic chemotherapy, the remission rate is not satisfactory (5, 6). Therefore, the identification of new immune-based molecular biomarkers is essential for clinical diagnosis, risk stratification, and treatment response prediction and monitoring (7, 8).

The tumor microenvironment is a crucial component of cancer (9). Metabolic reprogramming of tumors in the microenvironment is a crucial mechanism for adaptation, which confers metabolic plasticity for cancer cells, improves cell survival and promotes unlimited proliferation within the microenvironment, which is characterized by hypoxia and nutrient deficiency (10). For example, it has long been known that elevated glutamine dissolution and aerobic glycolysis are typical metabolic characteristics of cancer cells (11). Recently, cancer-specific lipid metabolic remodeling has attracted widespread attention (12). The features of increased exogenous lipid and lipoprotein intake, as well as overactivated ab initio synthesis, indicate that BRCA cells have a greater affinity for lipids and cholesterol than normal cells do. These factors directly contribute to the malignant transformation and progression of cancer cells, as well as the aberrant accumulation of lipids in the tumor microenvironment. Accordingly, these common lipid compounds also have an impact on tumor-associated immune cells that reside in the microenvironment (13). According to recent research, abnormalities in lipid metabolism within BRCA cells can hinder the stimulation, penetration and effectiveness of immune cells. This promotes immune escape and affects multiple aspects of the immune response (14). Additionally, drug resistance caused by abnormal lipid metabolism in BRCA has become a major obstacle to clinical treatment (15, 16). Therefore, the identification of prognostic genes associated with fatty acid metabolism may provide a viable therapeutic strategy for treating BRCA.

In this work, we constructed a LMPGS model pertaining to fatty acid metabolism, utilizing the TCGA BRCA cohort for training. The prognostic relevance of this model was subsequently validated in gene expression omnibus (GEO) datasets (GSE88770 and GSE20711). Furthermore, we investigated the associations between immunological responses and prognostic genes involved in the metabolism of fatty acids and the immune microenvironment as well as somatic mutations in patients. Additionally, single-cell pseudotime analysis revealed insights into the functions of these prognostic genes in tumor occurrence and development. Potential medications for the treatment of BRCA were predicted using molecular docking technology. In summary, LMPGS holds promise as a potential biomarker in human BRCA research, offering new perspectives for diagnosis and treatment.

## 2 Materials and methods

### 2.1 Data download and collation

We used the UCSC Xena Browser (https://xenabrowser.net/datapages/) (17) to download the TCGA-BRCA dataset in the transcriptome fragments per kilobase million (FPKM) format; this dataset contains survival data and corresponding clinicopathological data for 1280 patients. The single nucleotide variant (SNV) data, copy number variation (CNV) data, methylation data, mutation count, MSI sensor data and fragment genomic change frequency information of the corresponding samples were downloaded from the cBioPortal (18) database.

In addition, we downloaded the GSE88770 (19) and GSE20711 (20) transcriptome chip data and the clinical information of BRCA patients from the GEO (21) database. The GSE88770 dataset was generated by sequencing via the [HG-U133_Plus_2] Affymetrix Human Genome U133 Plus 2.0 Array. A total of 117 human BRCA tumor samples were selected as the validation set, and quality control steps were applied to exclude samples with low quality (based on background noise and signal intensity). The GSE20711 dataset was also generated by sequencing via the [HG-U133_Plus_2] [HG-U133_Plus_2] Affymetrix Human Genome U133 Plus 2.0 Array, which contains information on 88 human BRCA tumor samples. Normal tissue samples from two patients were included as controls to analyze gene expression differences.

In addition, we obtained the datasets from GSM5457205 (22), which included information on human BRCA tumor samples. The Illumina NovaSeq 6000 (Homo sapiens) platform was used to generate the sequencing data, and initial data processing included quality control steps, such as filtering out low-quality reads and performing adapter trimming. We further obtained data from MSigDB (https://www.gsea-msigdb.org/gsea/msigdb) (23). The online database includes 742 genes associated with lipid metabolism, and functional enrichment analysis will be performed to explore the biological significance of these genes in the context of BRCA.

### 2.2 Single-cell data processing

We used single-cell count data from the original UMI. In addition, Seurat v4.0 was used for preprocessing steps, quality control, normalization, dimensionality reduction clustering and clustering. Specific quality control criteria were established to ensure data integrity. First, every gene had to be expressed in a minimum of three cells, and each cell was required to expressed at least 200 genes. Second, the genes were selected based on the number of expressed genes in each sample, using the median ± 3*MAD (median absolute deviation) standard for filtering. Moreover, thresholds of 10% for the proportion of mitochondrial gene proportion and 1% for the proportion of hemoglobin gene proportion were set according to different sample types to exclude potential low-quality or dying cells. After applying quality control measures, we processed with data analysis using the Seurat package’s default parameters and standard operating procedures, which included: standardization of counts, normalization of data, identification of highly variable genes, dimensionality reduction (using PCA), and clustering of cells. The Harmony package was utilized to integrate data from multiple samples, thus correcting for batch effects. The annotation of single-cell groups was performed both manually and with the assistance of the scType package (23). Finally, the FindAllMarkers function used the Wilcoxon signed-rank test to compute differential gene expression between clusters, thereby identifying marker genes for each cell type.

### 2.3 Differential expression analysis

On the basis of the lipid metabolism genes in the MSigDB database, we performed gene scoring on the BRCA tumor tissue samples in the TCGA-BRCA dataset, and used the gene set variation analysis (GSVA) method to perform unsupervised scoring on each sample by gene set. The samples were subsequently divided into two groups according to the score ranking according to the GSVA score: the samples with the top 20% score were the Score+ group, and the samples with the bottom 20% score were the Score- group. We analyzed the DEGs in the TCGA-BRCA dataset between lipid metabolism score groups in human BRCA tumor samples using the rank-sum test.

### 2.4 Model construction and efficiency test

We used univariate Cox regression based on TCGA-BRCA data to screen genes associated with survival from DEGs grouped by lipid metabolism score. We integrated nine machine learning algorithms into 184 machine learning algorithm combinations, including LASSO, Ridge, elastic network (Enet), StepCox, survival support vector machine (survivalSVM), CoxBoost, supervised principal components (SuperPC), random survival forest (RSF), and generalized boosted regression modeling (GBM), to develop prognostic models.

The specific algorithm and parameters are as follows: (a) LASSO achieves feature selection through L1 regularization, with a regularization intensity λ set to 0.1, demonstrating excellent feature selection ability while effectively balancing model complexity and data fitting. (b) Ridge utilizes L2 regularization with a λ value of 0.1, which enhances robustness on the training set, ensuring model stability and adaptability to high-dimensional data while mitigating potential overfitting issues. (c) Enet integrates both L1 and L2 regularization techniques, making it suitable for scenarios where the number of features exceeds the number of samples. The α value is determined as 0.4 based on cross-validation results, enabling effective handling of multicollinearity problems by properly combining the advantages of LASSO and Ridge methods. (d) StepCox regression adopts the “direction=forward” method for feature selection based on the Cox proportional risk model. This progressive variable addition approach ensures focus on influential key genes in constructing a final model that can effectively identify important variables. (e) The survivalSVM possesses capabilities in dealing with survival time and event states using default parameter settings for model training purposes. With extensive application experience in survival analysis field, this algorithm generally captures complex patterns within survival data quite well. (f) CoxBoost employs Boosting concept to iteratively enhance the Cox model by utilizing a combination of 10 trees aiming at optimizing model fitting effect without excessive complexity introduction. (g) The SuperPC utilized default parameter settings to generate a linear combination of relevant features that captures the direction of greatest variation in the dataset. Optimal threshold evaluation was performed using cross-validation results, and application of the “pre-validation” function prevented issues with fitting multivariate Cox regression models to validation datasets. (h) RSF classification and regression, combining multiple decision trees with n_estimators set at 100 and default settings is widely accepted as it provides sufficient decision trees for increased prediction accuracy while ensuring model stability. (i) GBM improves model generalization by combining multiple weak learning models (usually decision trees) with a learning rate between 0.1-0.3 and tree depth set at 5 based on step-by-step experimentation and observation to ensure gradual growth without compromising stability.

After the construction and tuning of these models, we used two datasets, GSE88770 and GSE20711, as validation sets, and the average C-index as the main evaluation index of the model. The combination algorithm that achieved the highest C-index was chosen as the ultimate model. In addition, we collected literature on prognostic model construction from the TCGA-BRCA dataset from September 1, 2022 to September 1, 2023 (24–28).During this time, we conducted a literature survey to ensure that all the latest research findings were covered and corresponding data were used to compare the performance of the models.

### 2.5 Clinical feature correlation, pathway enrichment and pancancer analysis

First, we used the rank-sum test to compare clinical features between patients with high and low lipid metabolism scores, and the results are shown as bars. We then obtained data from the MSigDB (23) (https://www.gsea-msigdb.org/gsea/msigdb) database to download 50 tumor-associated pathways for gene set enrichment analysis (GSEA) (24, 29)to investigate the distinctions between groups with high and low scores in lipid metabolism. The transcriptome data and clinical characteristics of 32 tumors were downloaded from UCSC Xena, and the best machine learning combination model was subsequently used to analyze the prognostic effect of the model score in different tumors one by one.

### 2.6 Transcriptomic analysis of the high- and low-lipid metabolism score groups

We examined the variations in the tumor mutation burden (TMB), SNV data, and CNV data. We then selected the most important genes that make up the lipid metabolism score model for analysis, and we analyzed the differences in the transcript levels and methylation levels of these genes and the effects of individual genes on survival. For the selection criteria of the key genes in the lipid metabolism scoring model, first, we used univariate and multivariate Cox regression analyses to screen genes, and the selection criterion was a P value less than 0.05. Second, among the genes screened, those with a relatively high risk ratio (HR) and biological significance were prioritized to ensure that these genes not only were statistically significant, but also played an important role in lipid metabolism processes. We also used a combination of multiple machine learning algorithms (such as LASSO, Ridge, and CoxBoost) to construct prognostic models, and evaluated the predictive efficacy of each model. Finally, we selected the combination algorithm that performed best in the training set and validation set to screen 21 genes as the gene set of the LMPGS model.

### 2.7 Differential expression of genes linked to immune checkpoint inhibitors and immunogenic cell death in groups with high and low lipid metabolism scores

We analyzed information on 26 immunogenic cell death-related genes and 47 immune checkpoint inhibitor (ICI)-related genes. We then compared the differences between the two classes of genes in the TCGA-BRCA transcriptome data to determine whether the immunotherapy response varied between groups with high and low lipid metabolism scores.

### 2.8 Response to immunotherapy and chemotherapy medications in groups with high and low lipid metabolism scores

To assess the immune response, we used the tumor immune dysfunction and exclusion (TIDE) tool (http://tide.dfci.harvard.edu/login/) (30). In addition, we integrated the transcriptome-level data of each cell line from the cancer cell line encyclopedia (CCLE), the clinical trials research platform (CTRP), the PRISM database and the area under curve (AUC) data of 981 drugs (31). The difference in the AUC between different lipid metabolism score groups and the correlation between the AUC and lipid metabolism were analyzed to further identify therapeutic drugs with different scores.

### 2.9 Molecular docking

To further analyze the drug of choice from the last set of experiments and the composition of lipid metabolism genes encoding proteins, we used the PubChem database (https://pubchem.ncbi.nlm.nih.gov/) to obtain information on drug and lipid metabolism, mainly protein structure files. Then, batch processing and AutoDock Vina v.1.2.2 were used for molecular docking to select the receptor and ligand pairs with the minimum binding energy.

### 2.10 Pseudotime analysis

To further analyze the differentiation status between subsets, we used the classical monocle2 package (30) to perform a pseudotime analysis of T-cell subsets. The single-cell data were processed by constructing monocle objects, normalizing, and filtering low-quality cells and other processes. Highly discrete genes were selected to reduce the dimensionality of the data via the DDRTree method, and then the data were subjected to pseudotime analysis of different types of cells and important genes.

### 2.11 Acquisition of key genes from the immunohistochemical data

We utilized data from the human protein atlas (HPA) (32) to corroborate the variations in key gene expression between human BRCA tissues and normal tissues.

### 2.12 Cell lines and cell culture conditions

The ATCC provided the MCF10A, MDA-MB231, BT549, and SUM149PT cells. MCF10A, MDA-MB231, BT549, and SUM149PT cells were acquired from the Type Culture Collection of the Chinese Academy of Sciences in Shanghai, China. Dulbecco’s modified Eagle’s medium (DMEM) supplemented with 10% bovine calf serum (HyClone) was used to culture MDA-MB231, and SUM149PT cells. Ten percent bovine calf serum (HyClone) was added to complete Roswell Park Memorial Institute 1640 (RPMI 1640) medium to culture the BT549 cells. DMEM/F12 supplemented with 10% bovine calf serum (HyClone) was used to culture the MCF10A cells. Every cell line was grown in a humidified incubator with 5% CO2 at 37°C.

### 2.13 qPCR

Follow the manufacturer’s guidelines to extract total RNA from cells using TRIzol (Invitrogen). Using an Applied Biosystems TaqMan reverse transcription reagent kit, 1 mg of RNA per sample was utilized to synthesize cDNA. Using the SYBR Premix Ex Taq kit for real-time PCR (TaKaRa), qRT−PCR was conducted using an Applied Biosystems 7500 real-time PCR system. The primer sequences are shown in **Supplementary Table S1**.

### 2.14 Immunohistochemistry

Samples of adjacent matched nontumor tissues and human BRCA tissues were acquired from the Affiliated Cancer Hospital of Harbin Medical University. IHC labeling was performed on nontumor and BRCA samples from 50 female subjects. The percentage of positive cells was used to assess tissue section staining quantitatively.

### 2.15 Western blot

The cells were lysed on ice with RIPA lysis buffer. The proteins were separated via SDS−polyacrylamide gel electrophoresis, transferred to a PVDF membrane, blocked with 5% skim milk powder and washed with PBST. The membranes were subsequently incubated with the primary antibody at 4°C overnight. The membranes were incubated with secondary antibody for 1 hour after washing with PBST, and the protein bands were detected with enhanced chemiluminescence (ECL) luminescent solution.

### 2.16 CCK8 assay

The cells were plated in 96-well plates at 3000 cells per well. After 24 hours, the control group was treated with DMSO, and the experimental group was treated with different concentrations of niclosamide dissolved in DMSO. Cell viability was assessed via CCK-8 kit, and the light absorption value was measured at 450 nm with microplate reader.

### 2.17 Antibodies and reagents

Antibodies against the following proteins were used for the IHC and western blot experiments: ACSL1 (Proteintech, 13989-1-ap, IHC: 1:200, western blot: 1:1000), ACSF2 (Proteintech, 16140-1-ap, IHC: 1:200, western blot: 1:1000), CPNE3 (Proteintech, 11186-1-ap, IHC: 1:200, western blot: 1:1000), MTMR9 (ABclonal, A13124, IHC: 1:200, western blot: 1:1000), and β-actin (Santa Cruz, western blot: 1:1000). The following reagents were used in the CCK8 experiments: niclosamide (CAS No. 50-65-7, MedChemExpress, BAY2353) and Cell Counting Kit-8 (LABLEAD, CK001).

### 2.18 Statistical analysis

In this work, R software (https://www.www.r-project.org/, version 4.2.1) was used for all data calculations and statistical analyses. If not otherwise specified, correlation analyses were performed via Spearman’s correlation analysis via the Hmisc function of the base package of R software. The Wilcoxon rank sum test was used to compare MSI scores, altered portions of the genome, and mutation counts between the Score+ and Score- groups. The C-index, cross-validation and model ranking were used to determine the best machine learning mix. We used the Wilcoxon rank sum test to compare differences in gene transcription and methylation levels between the high-rated and low-rated groups. To control the error finding rate of multiple tests, we also adopted the Benjamini−Hochberg correction method. In addition, the differences of immune-related gene expression and immunoinfiltration indexes between the high-low rating groups were statistically analyzed via the Wilcoxon rank sum test. All of the statistical P values were bilateral, and differential gene screening was statistically significant if the corrected p value was < 0.05 (ns stands for p value>0.05; * stands for 0.01< p value <0.05; ** stands for 0.001< p value <0.01; *** stands for p value <0.001; **** stands for p value <0.0001).

## 3 Results

### 3.1 GSVA and genomic differences between lipid metabolism groups

**Figure 1:**
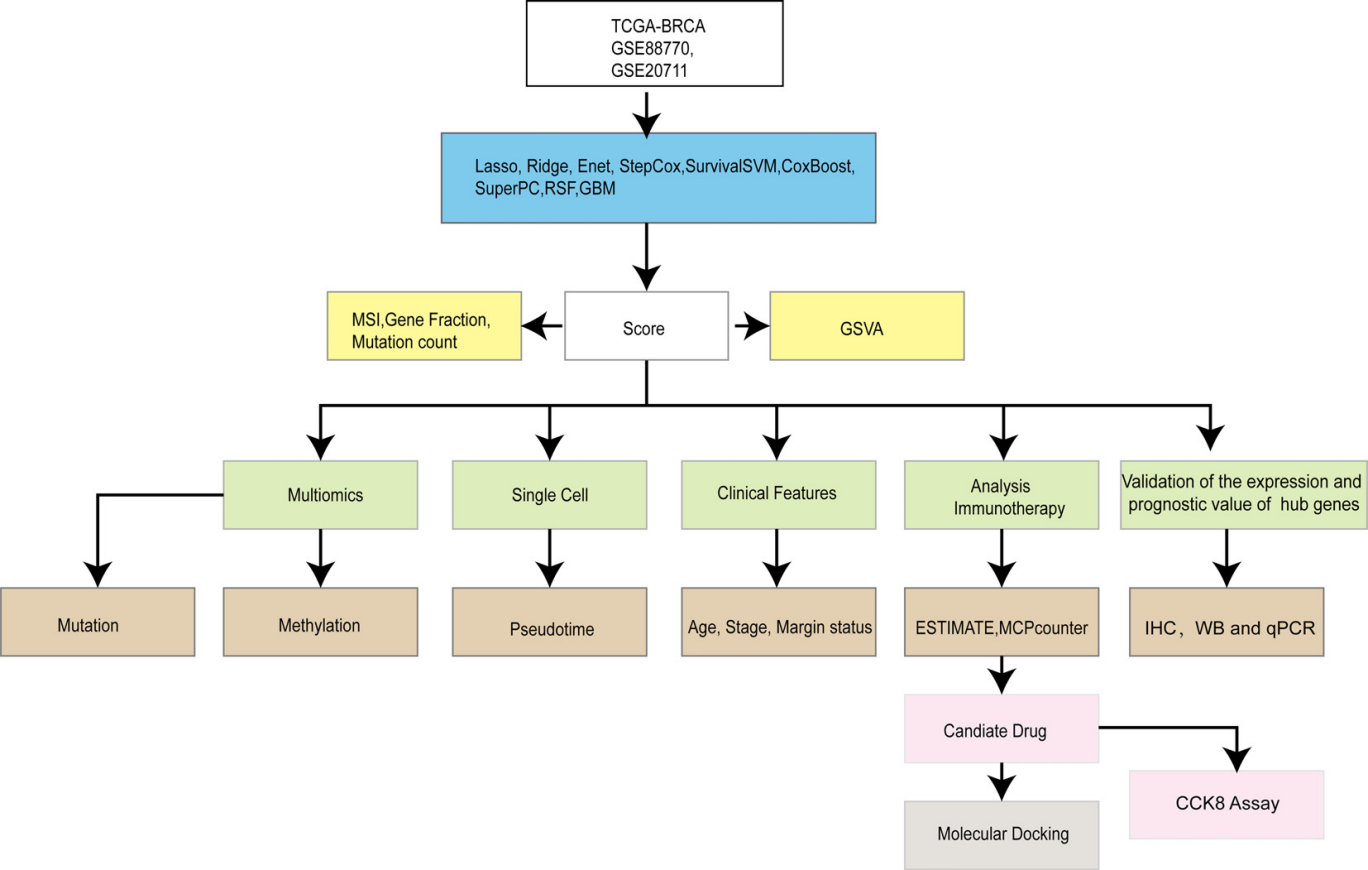
Overall design idea.

**Figure 2:**
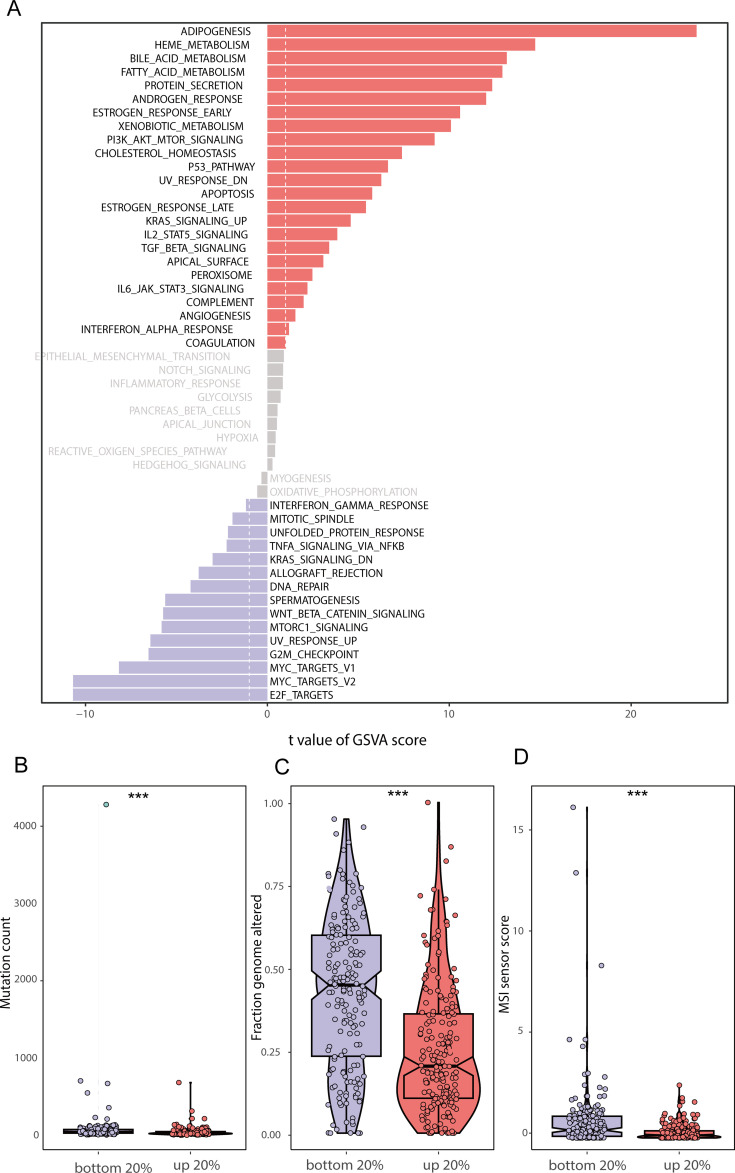
GSVA enrichment analysis and genomic differences between lipid metabolism groups **(A)** GSVA enrichment results between the high and low lipid metabolism score groups; **(B)** Difference in mutation counts between the top 20% and the bottom 20% of samples with lipid metabolism score; **(C)** Frequency of segmental genomic alterations between the top 20% and bottom 20% of the lipid metabolism scores; **(D)** Difference between MSI sensor scores between the top 20% and the bottom 20% of samples with lipid metabolism scores. (*** stands for p value <0.001).

Our overall experimental design is shown in **Figure 1**. We scored human BRCA tumor tissue samples in the TCGA-BRCA dataset based on the lipid metabolism genes obtained from the MSigDB database. We selected the top 20% of the samples as the Score+ group and the bottom 20% of the samples as the Score- group. First, we performed GSVA separately for the Score+ group and the Score- group (**Figure 2A**), where a t value of GSVA greater than 1 was taken as the cut-off value. We found that the samples in the Score+ group were significantly enriched in the Adipogenesis, Heme metabolism, Bile acid metabolism, and Fatty acid metabolism gene sets. The Score- group was significantly enriched in the E2f target, Myc target v2, Myc target v1 and G2M checkpoint gene sets. On the basis of this grouping, we compared whether the microsatellite instability (MSI) score (**Figure 2D**), fraction of the genome altered (**Figure 2C**) and mutation count (**Figure 2B**) differed between the two groups. We found that the MSI score, fraction of the genome altered and mutation count in the Score- group were noticeably greater than those in the Score+ group.

### 3.2 Grouping of TCGA-BRCA tumor samples according to lipid metabolism score

**Figure 3:**
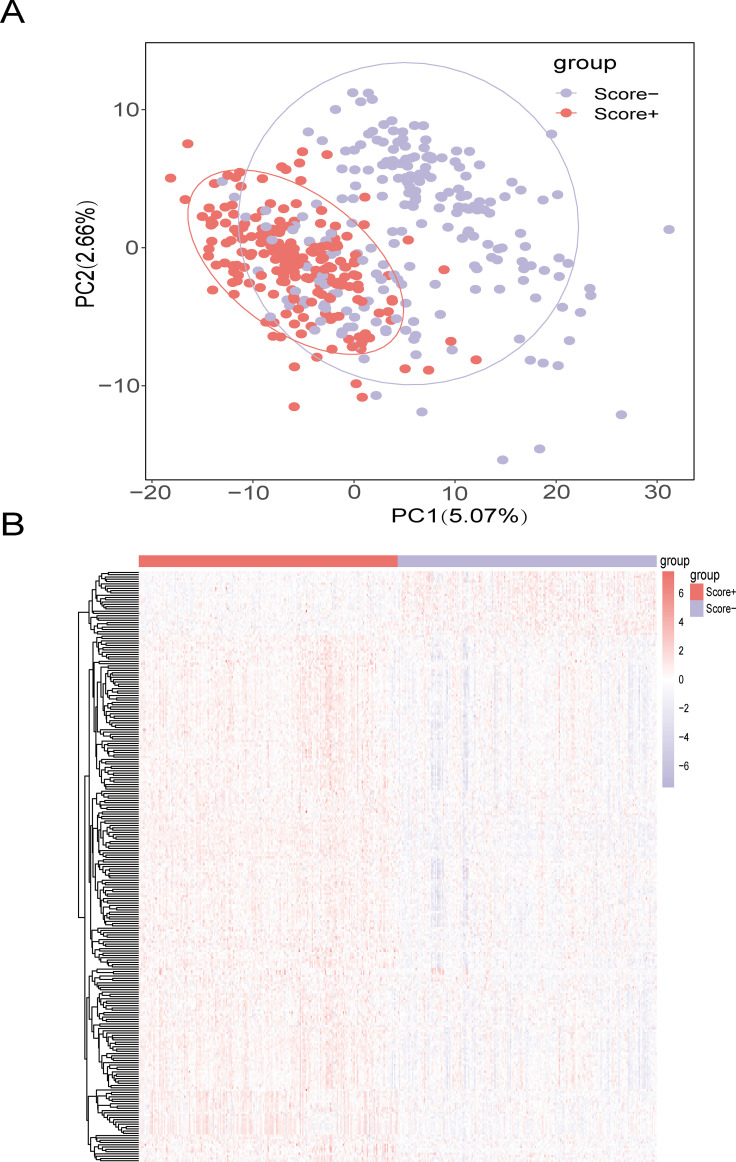
Screening of DEGs between the high- and low-lipid metabolism score groups **(A)** Principal component analysis (PCA) between high and low lipid metabolism score groups in the TCGA-BRCA training set; **(B)** Heatmap of DEGs between the high- and low-lipid metabolism score groups in the TCGA-BRCA training set. The color bars from red to blue indicate gene expression from high to low, with red indicating high expression and blue indicating low expression.

We selected the top 20% of the samples as the Score+ group and the bottom 20% of the samples as the Score- group. The Score+ group included 217 samples, and the Score- group also included 217 samples. We used the rank-sum test to analyze differences between the high- and low-lipid metabolism groups. To screen for significant DEGs, we set the thresholds to |logFC| > 0.5 and adjusted P value < 0.05; ultimately, we identified 259 genes related to lipid metabolism that were differentially expressed between the Score+ and Score- (high- and low-score) groups (**Figures 3A, B**).

### 3.3 Construction of prognostic models related to lipid metabolism

**Figure 4:**
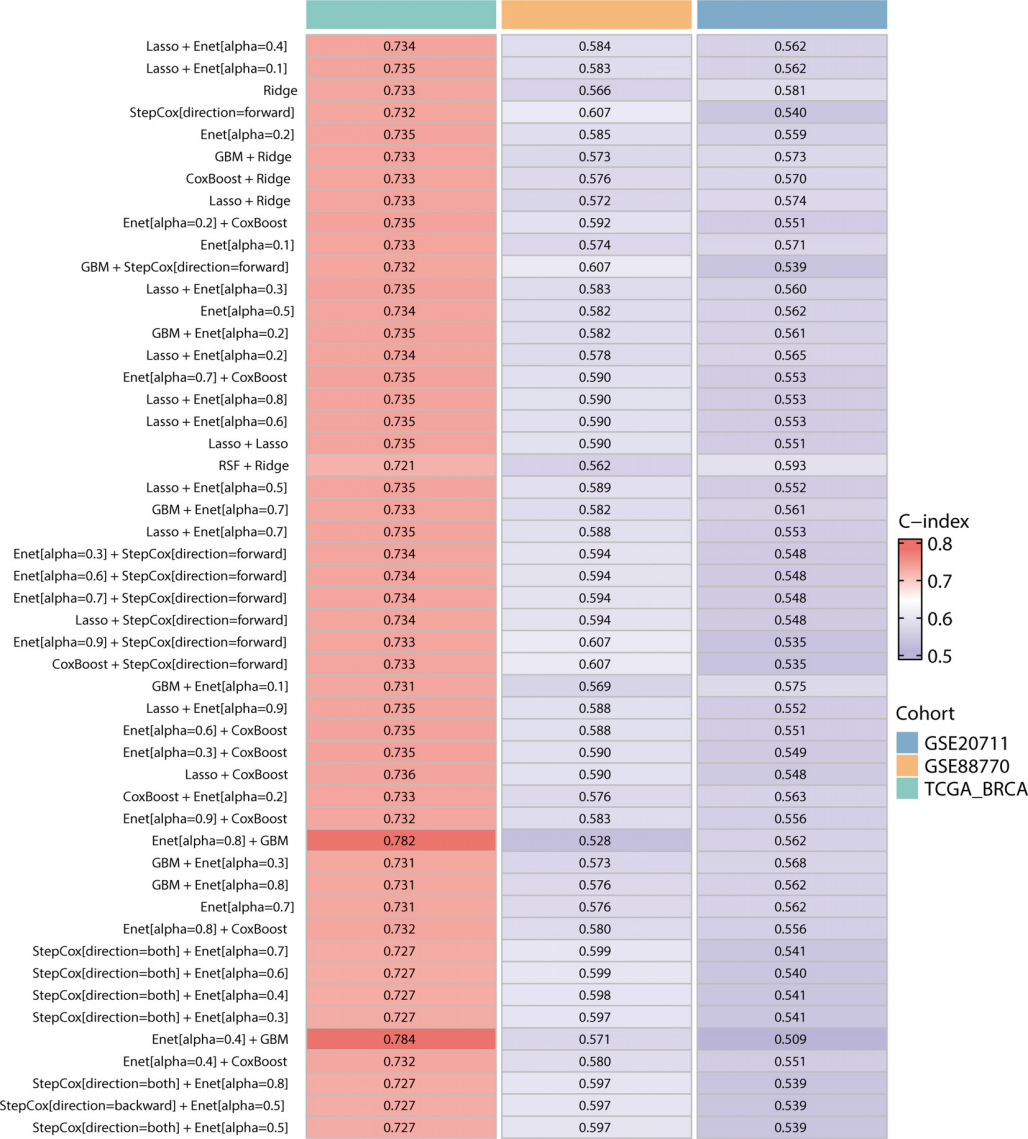
Construction and validation of the prognostic models in the TCGA-BRCA, GSE207711 and GSE88770 datasets. The top 50 machine learning combination algorithms for the average C-index in the TCGA-BRCA training set and the GSE20771 and GSE88770 validation sets. We used the prediction function to calculate the elastic network (alpha=0.4) combined with LASSO for LMPGS, a score consisting of 21 genes (ACAA1, ACSF2, ACSL1, ALOX15, ALOX15B, APOA5, CPNE3, CPT1A, CYP2D6, CYP4F11, ENPP6, FABP7, GSTM4, INSIG2, LIPH, MBTPS2, MTMR9, OSBPL10, PRKAA2, SLC27A2, STAR), of which the three most important genes were ACSF2, MTMR9, and ACSL1.

To construct a prognostic model focused on lipid metabolism-related genes, we initially employed univariate Cox regression analysis to pinpoint 26 genes associated with prognosis from the pool of 259 DEGs (p value < 0.05). Then, we utilized a combination of machine learning algorithms. We carried out parameter tuning for each basic algorithm, and different combinations were generated under different parameter settings. As seen from the specific combinations listed on the left side of **Figure 4**, not only were there multiple parameter combinations for each individual algorithm, but pin-to-pair combinations and even multiple combinations between algorithms were also explored. Through these parameter adjustments, different ratio feature selection strategies, cross-validation strategies, and the use of integration methods, 184 model combinations were generated from 9 basic machine learning algorithms. Next, we evaluated the performance of 184 combinations of the machine learning algorithms using two datasets, GSE88770 and GSE20711, as validation sets. The C-index was used as the main model evaluation metric. The results showed that the machine learning combination of the elastic network (alpha=0.4) combined with LASSO had the highest C-index, so we selected this model as the best model (**Figure 4**). Notably, TCGA-BRCA has a larger C index than the other two validation sets do, which may be related to the larger data volume and low noise of the data, and the greater amount of clinical information contained in it may provide additional context for the model, further enhancing its predictive power.

### 3.4 Validation of lipid metabolism-related prognostic models

**Figure 5:**
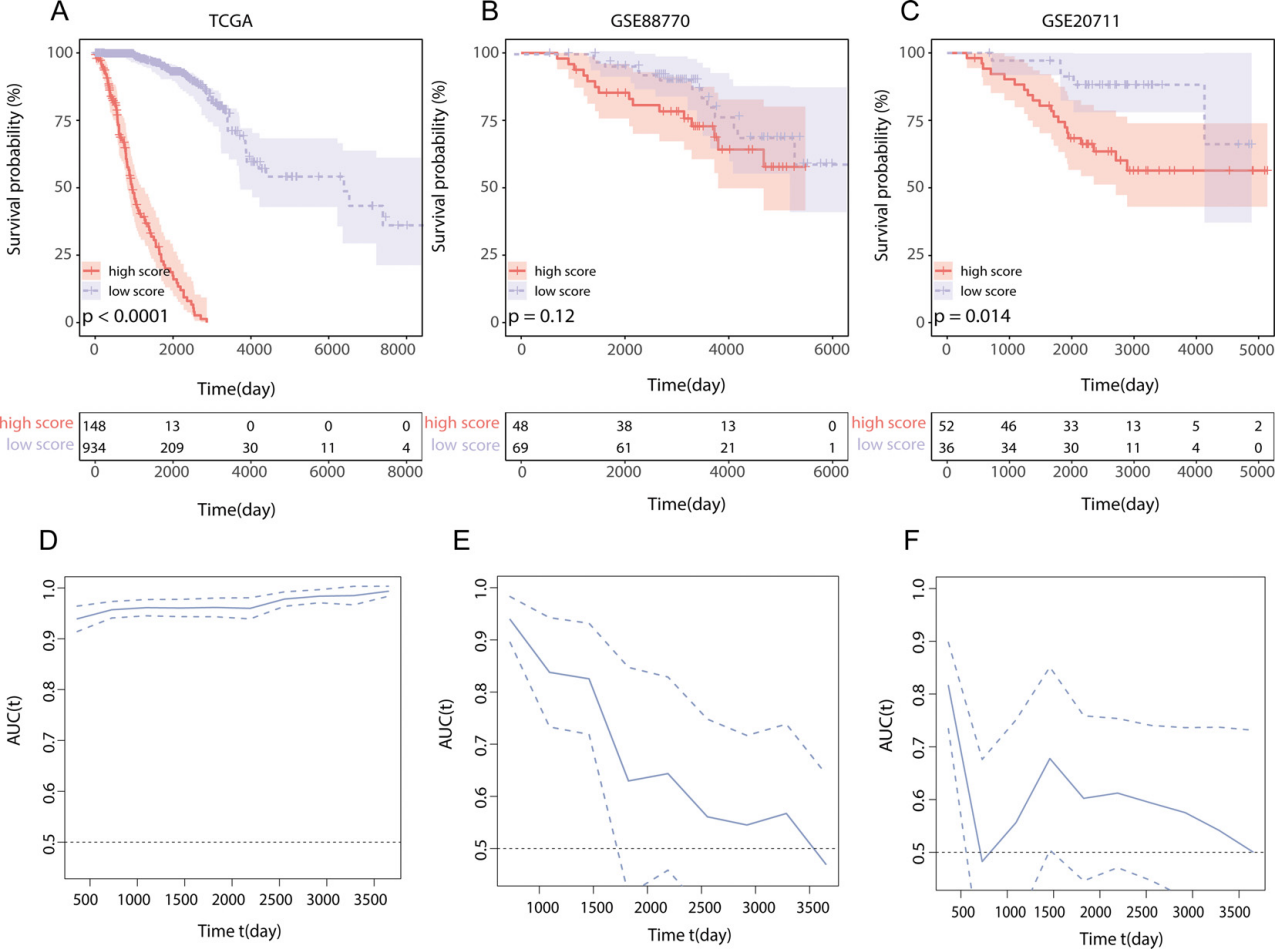
Effect of LMPGS on OS and validation of time−ROC curves in TCGA-BRCA, GSE88770, and GSE20711 **(A)** Survival difference between high and low score groups of LMPGS in the TCGA-BRCA training set; **(B)** Survival difference between high and low score groups of LMPGS in GSE88770 validation dataset; **(C)** Survival difference between high and low score groups of LMPGS in GSE20771 validation dataset; **(D)** Line plot of AUC of the training set TCGA-BRCA for BRCA OS at different time points; **(E)** Line plot of AUC of test set GSE88770 on BRCA OS at different time nodes; **(F)** Line plot of AUC of test set GSE20711 on BRCA OS at different time points.

To assess the influence of this score on the overall survival (OS) of BRCA patients, we utilized the optimal cutoff value to categorize patients into two groups according to the LMPGS. The group with a high score for LMPGS had a notably lower OS rate than did the group with a low score in the training set (**Figure 5A**, p value < 0.0001). The same conclusion was drawn for the GSE88770 and GSE20711 validation sets (**Figure 5B**, p value = 0.12; **Figure 5C**, p value =0.014). To further validate the precision of the prognostic model, time−ROC curves and Kaplan−Meier (KM) survival curves were plotted for each of the above three datasets. We found that the AUC was greater than 0.9 in the TCGA-BRCA cohort, greater than 0.6 in the GSE88770 test cohort for the first 5 years, and greater than 0.6 in the GSE20711 test cohort for most of the first 10 years (**Figures 5D–F**).

### 3.5 Comparison of lipid metabolism-related prognostic gene score models and other published score models for predicting overall survival in BRCA patients

**Figure 6:**
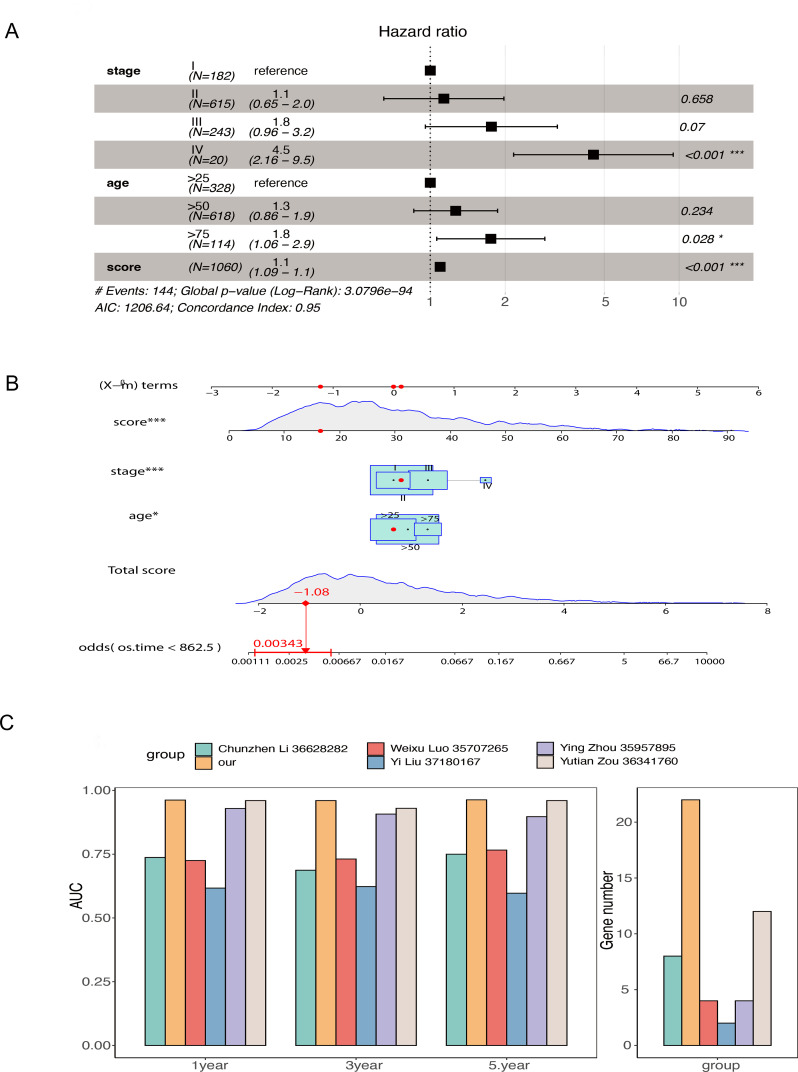
Comparison of the overall model including prognostic genes related to lipid metabolism and other models constructed from TCGA-BRCA data **(A)** Forest plot of multivariate Cox regression results in TCGA-BRCA data; **(B)** Nomogram model of prognostic genes related to lipid metabolism was constructed; **(C)** Comparison of AUC and number of genes at 1, 3 and 5 years between the overall model of prognostic genes related to lipid metabolism and other models collected by retrieval (* stands for 0.01< p value <0.05; *** stands for p value <0.001).

To compare the accuracy of our lipid metabolism-related prognostic gene score model and other score models in predicting overall prognosis in BRCA patients, we first performed a multivariate analysis including the lipid metabolism-related prognostic gene score and other clinicopathological features using the TCGA-BRCA dataset. The lipid metabolism-related prognostic gene score, age, and tumor stage were identified as prognostic factors for BRCA patients (**Figure 6A**). A nomogram model was constructed (**Figure 6B**), and we employed a multivariate Cox regression model to assess the prognostic lipid metabolism-related gene score, age, and tumor stage. We also conducted a literature survey and searched for articles from September 1, 2022, to September 1, 2023, with keywords such as TCGA, prognosis and BRCA, and a total of 5 relevant articles were collected. We combined the AUC scores of these models and the number of genes included. In the comparison of the 1-, 3-, and 5-year survival data, our model had the highest AUC of the multiple models, and at the same time, the number of genes included in our model was relatively large (**Figure 6C**).

### 3.6 Differences in clinical characteristics and pathway enrichment between the high- and low-score groups based on prognostic lipid metabolism-related genes and their application across cancers

**Figure 7:**
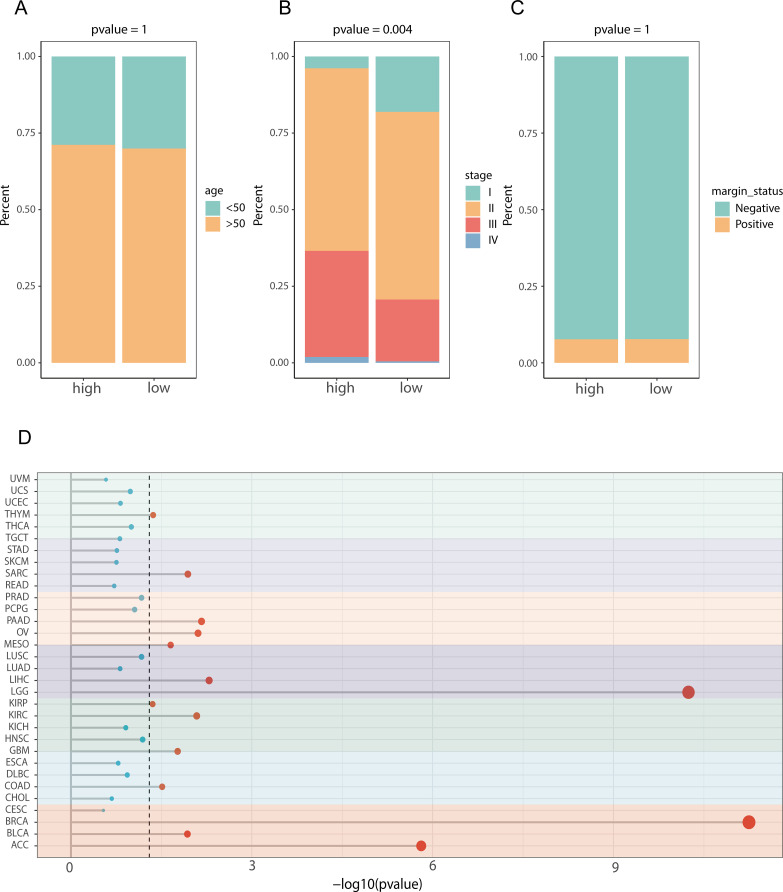
Clinical characteristics and pathway differences between the high and low lipid metabolism-related prognostic gene score groups in the TCGA dataset and their application across cancers. **(A)** Age difference between the high- and low-score groups in terms of prognostic genes related to lipid metabolism; **(B)** The difference of tumor stage between the high- and low-lipid metabolism-related prognostic gene score groups; **(C)** Difference in tumor status between the high- and low-lipid metabolism-related prognostic gene score groups; **(D)** Effect of LMPGS on the OS of all tumors in the TCGA database.

To explore the clinical distinctions between groups characterized by high and low scores for prognostic genes linked to lipid metabolism, we initially conducted a comparative analysis of age (**Figure 7A**), tumor stage (**Figure 7B**), and tumor status (**Figure 7C**). We found a significant difference in tumor stage (p = 0.004) between the high- and low-score groups founded on LMPGS model, whereas no notable disparities were observed in age or tumor status between these groups. We next assessed the effect of the lipid metabolism-related prognostic gene score on OS in patients with 31 additional tumor types. The results revealed that the lipid metabolism-related prognostic gene score was associated with OS in adrenocortical carcinoma, bladder urothelial carcinoma, cholangiocarcinoma, colon cancer, and other tumors, suggesting that the lipid metabolism-related prognostic gene score on OS has broad applicability (**Figure 7D**).

### 3.7 Transcriptomic variations between patients sorted into high- and low-score groups according to the expression of prognostic genes related to lipid metabolism

**Figure 8:**
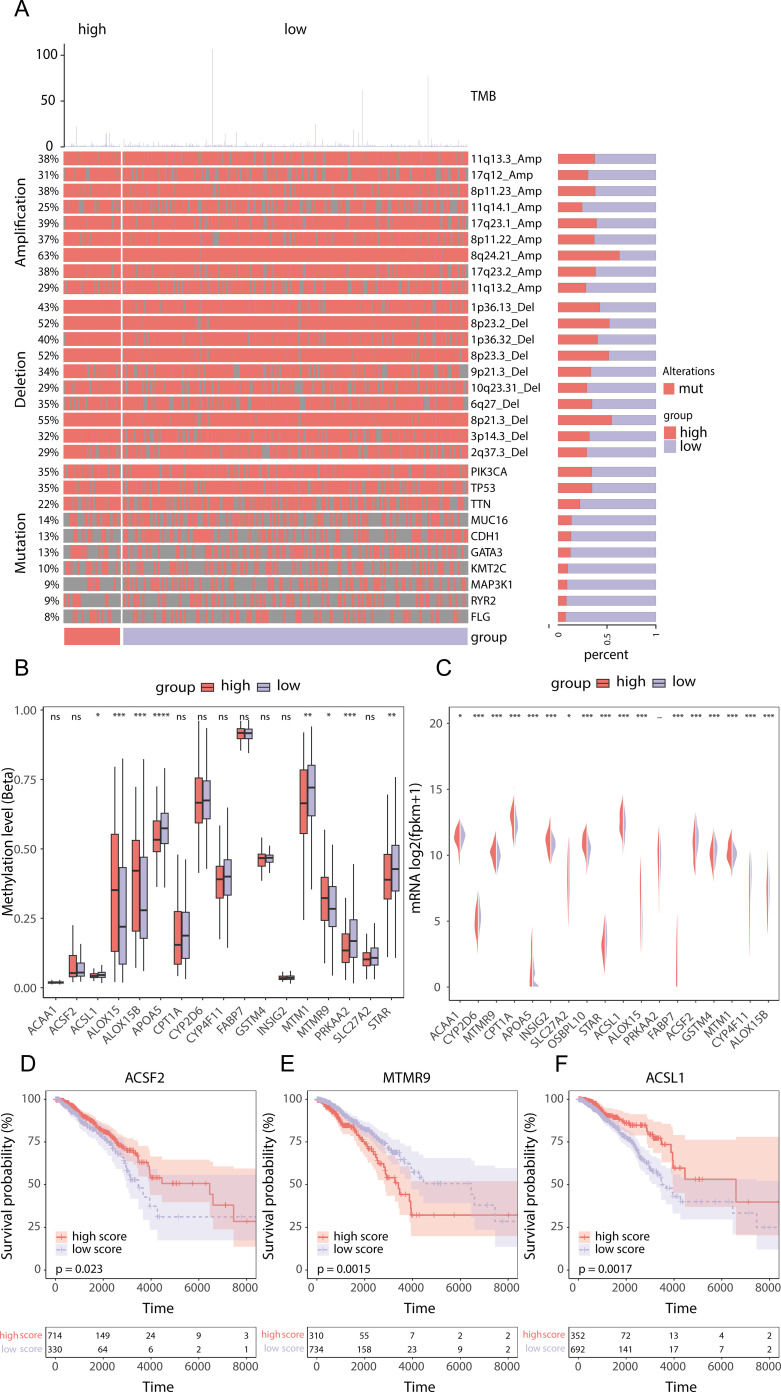
Transcriptomic differences between the high- and low-score groups of prognostic genes related to lipid metabolism in the TCGA-BRCA dataset. **(A)** Genomic expression differences between the high- and low-score groups of prognostic genes related to lipid metabolism; **(B)** Differences in the methylation levels of prognostic genes related to lipid metabolism; **(C)** Transcriptome level differences in LMPGS; **(D)** Correlation of ACSF2 with BRCA OS; **(E)** Correlation of MTMR9 with BRCA OS; **(F)** Correlation of ACSL1 with BRCA OS. (ns stands for p value>0.05; * stands for 0.01< p value <0.05; ** stands for 0.001< p value <0.01; *** stands for p value <0.001;**** stands for p value <0.0001).

To further understand the differences between patients with high and low scores, we performed a transcriptomics analysis. First, we compared genomic variations to compare the TMB and mutation, deletion, and amplification profiles between the two groups (**Figure 8A**). Next, we focused on determining the differences between the two groups in terms of the three most important genes that composed the prognostic model of genes. In terms of methylation levels, the methylation level of ACSL1 was significantly greater in the low-score group (p value<0.01), while the methylation level of MTMR9 was greater in the high-score group (p value<0.01), and the methylation level of ACSF2 was not significantly different between the two groups (**Figure 8B**). At the transcriptome level, ACSL1, ACSF2 and MTMR9 were highly expressed in the high-score group (**Figure 8C**). In addition, the transcriptome expression levels of these three genes were also significantly correlated with the OS of BRCA patients. Compared with those of the low-expression group, the survival rates of the high-expression groups of ACSF2 (**Figure 8D**, p value=0.023) and ACSL1 (**Figure 8F**, p value=0.0017) were significantly better. Patients in the low-MTMR9 expression group had a considerably better survival rates than those in the high-MTMR9 expression group (**Figure 8E**, p value=0.0015).

### 3.8 Differences in immune cell infiltration between patients grouped into high- and low-score groups according to the levels of genes related to lipid metabolism and prognosis

**Figure 9:**
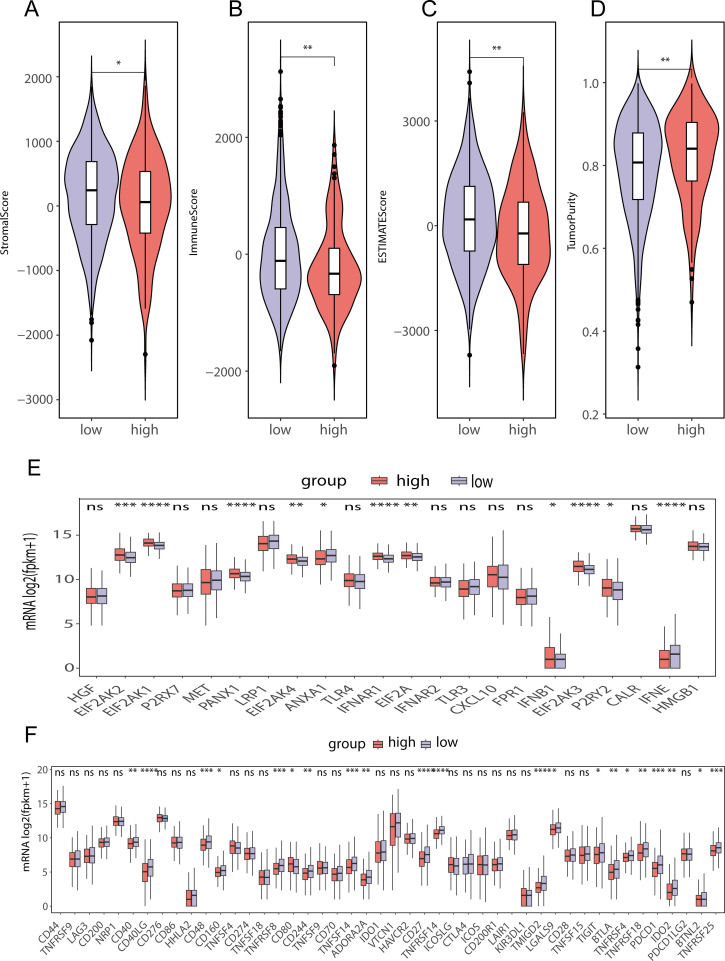
Differences in immune cell infiltration between the high- and low-lipid metabolism prognostic score groups in the TCGA-BRCA data **(A)** ESTIMATE stromal score differences between high- and low-score groups of prognostic genes related to lipid metabolism in TCGA-BRCA data; **(B)** ESTIMATE immunoscore differences between high- and low-score groups of prognostic genes related to lipid metabolism in TCGA-BRCA data; **(C)** ESTIMATE score differences between high- and low-score groups of prognostic genes related to lipid metabolism in TCGA-BRCA data; **(D)** ESTIMATE differences in tumor purity between high- and low-score groups of prognostic genes related to lipid metabolism in TCGA-BRCA data; **(E)** Differences in immunogenic cell death-related genes between high- and low-lipid metabolism-related prognostic gene score groups in TCGA-BRCA data; **(F)** Difference of ICI-related genes between high- and low- score groups of LMPGS in TCGA-BRCA data. (ns stands for p value>0.05; * stands for 0.01< p value <0.05; ** stands for 0.001< p value <0.01; *** stands for p value <0.001;**** stands for p value <0.0001).

To assess the differences in immune cell infiltration between the two score groups, we used the ESTIMATE method (**Figure 9C**). The findings indicated that the group with a low lipid metabolism-related prognostic gene score had higher stromal (**Figure 9A**) and immune (**Figure 9B**) scores, while the high lipid metabolism-related prognostic gene score group of had greater tumor purity (**Figure 9D**). These findings suggest that the low-score group may exhibit a greater degree of immune cell infiltration than the high-score group does. Moreover, we further analyzed the differences in immunogenic cell death-related genes between the lipid metabolism-related prognostic gene score groups and found that ANXA1, IFNE, LRP1 and other genes were highly expressed among the LMPGS in the low-score group. CALR, EIF2AK1, EIF2AK1 and other genes were highly expressed in the LMPGS high -score group, and the majority of genes linked to immunogenic cell death differed between the two groups (**Figure 9E**). Next, we examined the differences in ICI-related genes between the prognostic gene groups associated with lipid metabolism. TNFRSF9, CD200, PDCD1, IDO1 and other genes were highly expressed in the low lipid metabolism-related prognostic gene score group, and the expression of approximately half of the ICI-related genes differed between the two groups (**Figure 9F**).

### 3.9 Efficacy of chemotherapy and immunotherapy and prediction of candidate drugs for patients with high- and low-scores according to the levels of lipid metabolism and prognosis-related genes

**Figure 10:**
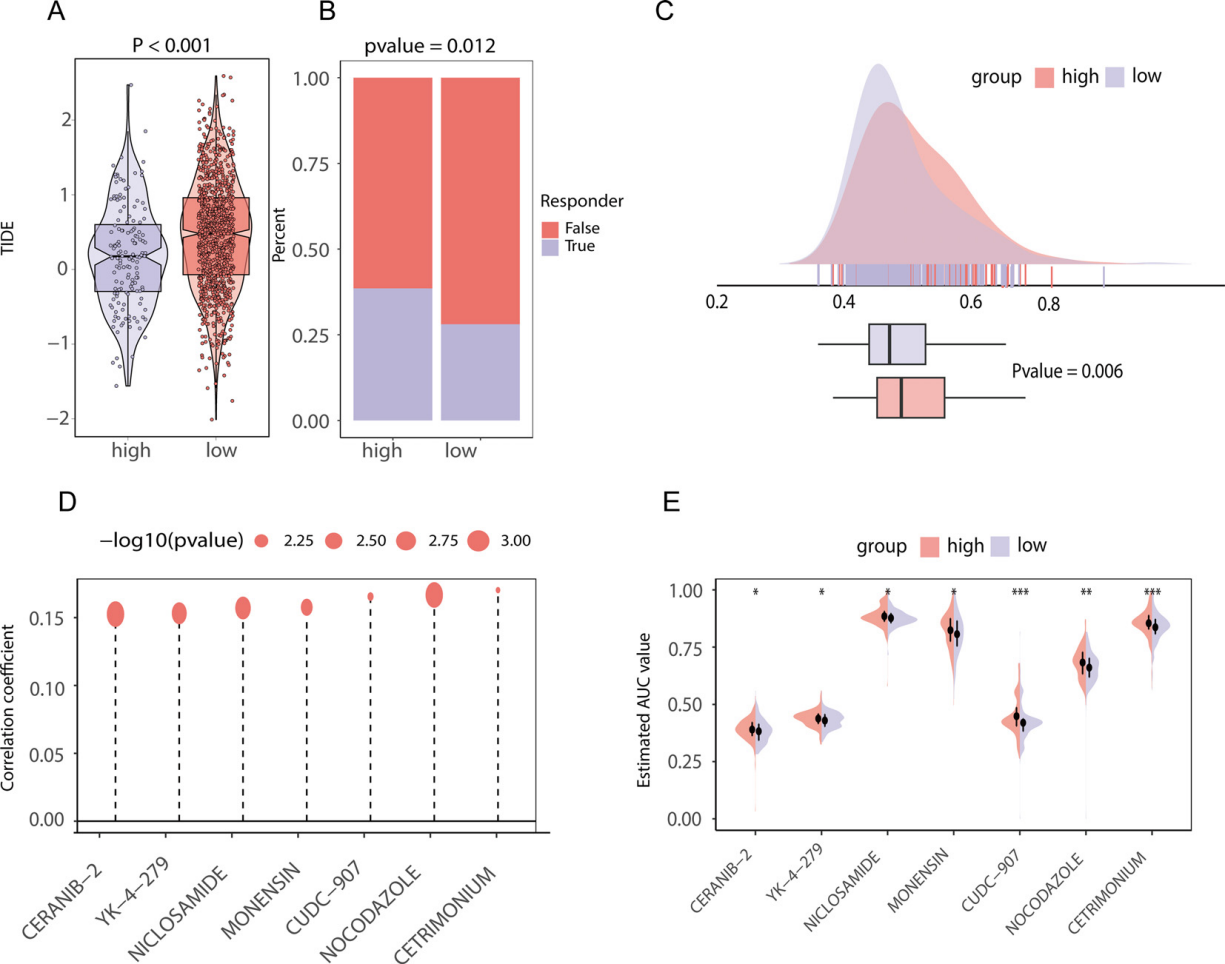
Chemotherapeutic and immunotherapy effects and drug prediction between high and low score groups of lipid metabolism prognostic genes in TCGA-BRCA data **(A)** TIDE score difference between the high- and low-score groups of lipid metabolism prognostic genes; **(B)** The proportion of patients who responded to immunotherapy in the high- and low-lipid metabolism prognosis related gene score groups; **(C)** Area under the drug-time curve of punabulin between the high- and low-lipid metabolism-related prognostic gene score groups. The higher area under the drug-time curve of the high rating group of lipid metabolism prognostic genes indicated that the group had better drug absorption of punabulim; **(D)** Drug lollipop plot associated with the lipid metabolism prognostic gene score; **(E)** Violin plot of drugs with the area under the drug-time curve difference between the high- and low-lipid metabolism prognostic gene score groups. (ns stands for p value>0.05; * stands for 0.01< p value <0.05; ** stands for 0.001< p value <0.01; *** stands for p value <0.001;**** stands for p value <0.0001).

The TIDE scores of the two groups varied, indicating that the two groups have different responses to immunotherapy (**Figure 10A**). In addition, the percentage of patients who responded to immunotherapy predicted by the TIDE database varied significantly (**Figure 10B**). To examine in more detail how groups with high and low lipid metabolism-related prognostic gene scores respond differently to chemotherapy, we used the first-line BRCA chemotherapy drug Plinabulin for validation. The findings indicated that the group scoring high exhibited greater AUC values, indicating good drug absorption (**Figure 10C**). Subsequently, we analyzed the drug data from the CCLE and PRISM databases and found that the sensitivity to 7 drugs (CERANIB-2, YK-4-279, NICLOSAMIDE, MONENSIN, CUDC-907, NOCODAZONE, and CETRIMONIUM) was significantly correlated with the lipid metabolism-related prognostic gene score (**Figure 10D**, p value <0.05). Moreover, there were notable differences in the dose−response curves between the two score groups (p value <0.05, **Figure 10E**).

### 3.10 Molecular docking

**Figure 11:**
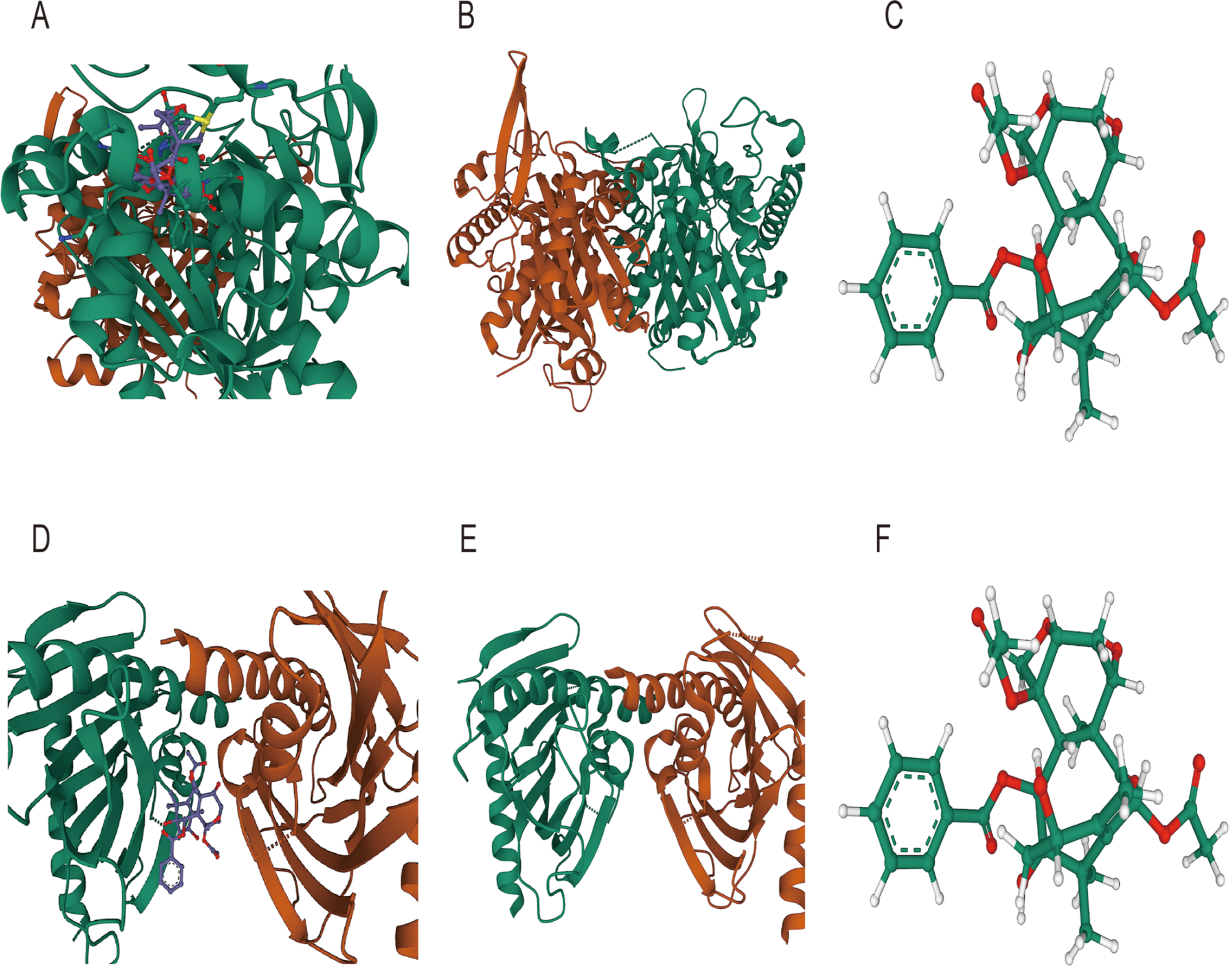
Molecular docking diagram **(A)** Molecular docking diagram between ACSF2 and niclosamide; **(B)** Structure of the ACSF2-encoded protein; **(C)** Ligand structure of niclosamide **(D)** Molecular docking diagram between MTMR9 and niclosamide; **(E)** Protein structure encoded by MTMR9; **(F)** Ligand structure of niclosamide.

To further examine the possibility of interactions between these seven possible medications and the proteins encoded by the three most significant genes (ACSF2, ACSL1, and MTMR9) in lipid metabolism-related prognostic gene set, we acquired structural information on the proteins produced by these three genes as well as the molecular structures of the pharmacological ligands from the PubChem database. We subsequently performed molecular docking via AutoDock Vina v.1.2.2 to identify receptor−ligand pairs with binding free energies less than -5 kcal/mol. The results showed that only ACSF2, MTMR9 and NICLOSAMIDE could bind to each other (**Figures 11A–F**). These findings may help elucidate the interactions between drugs and target proteins and provide important clues for further research and drug design.

### 3.11 Single-cell analysis

**Figure 12:**
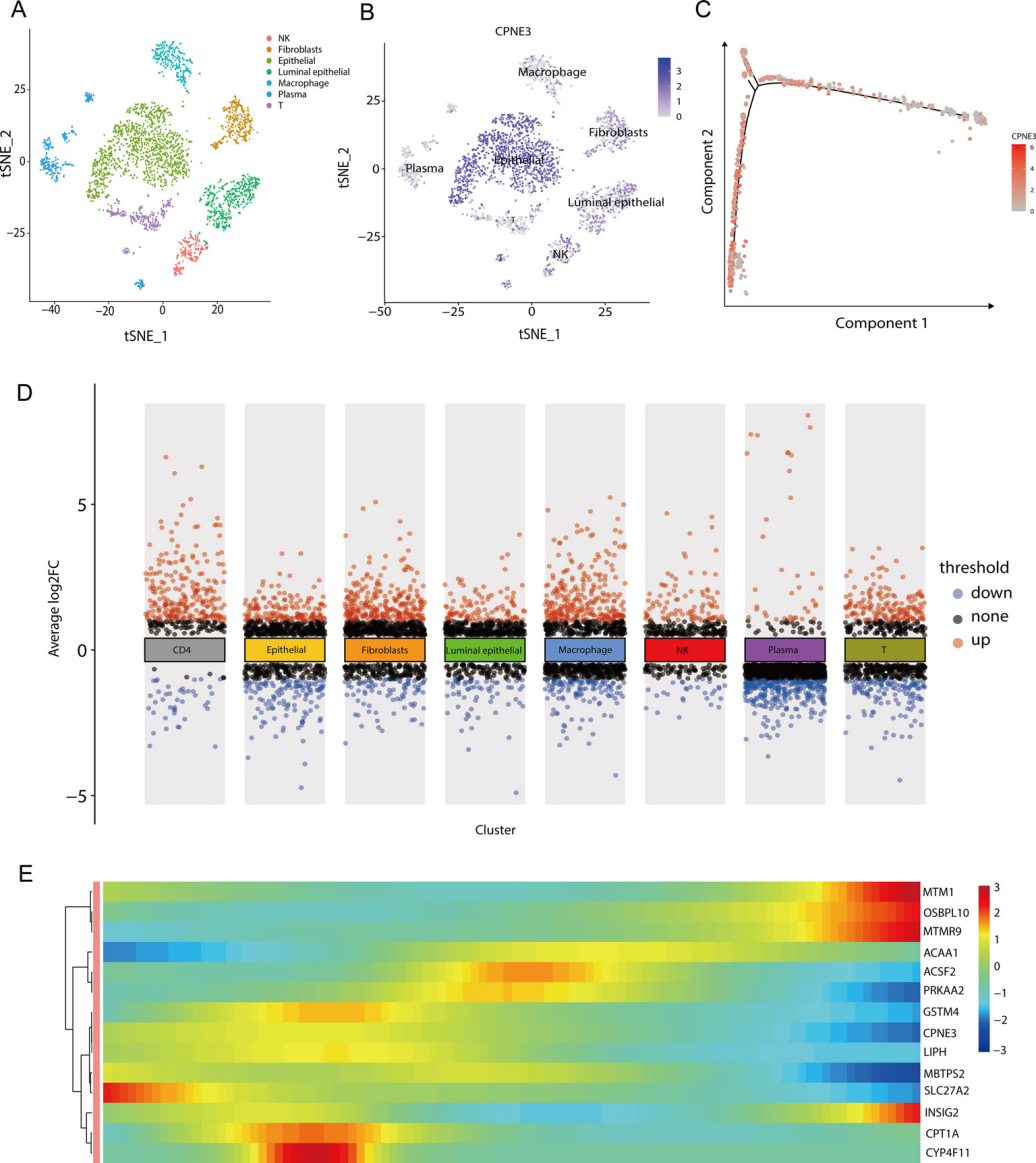
Grouping and differential analysis and pseudotime series analysis of single-cell data **(A)** When the resolution was 0.8, the GSM545720 dataset was divided into eight cell populations; **(B)** The expression of CPNE3 in each cell population, the darker the color, the higher the expression in the cell population; **(C)** Changes in the expression of CPNE3 over time. As time goes on, the red color gradually becomes lighter, representing a gradual decrease in the expression amount; **(D)** DEGs among various cell populations, among which CPNE3, as one of the genes related to lipid metabolism and prognosis, is highly expressed in epithelial cells; **(E)** Changes in the expression of genes related to lipid metabolism prognosis over time. The color bar from red to blue represents the gene expression from high to low.

We performed quality control of the GSM545720 BRCA dataset according to quality control standards and then performed dimensionality reduction and clustering. We subsequently set a resolution value of 0.8 as the clustering criterion and collected the signature markers of each cluster from published single-cell studies. Finally, we identified eight subpopulations: epithelial cells, endotheliocytes, fibroblasts, NK cells, T cells, plasma cells, CD4 cells, and macrophages (**Figure 12A**).

We subsequently performed differential expression analysis via the FindAllMarkers function using the Wilcoxon method and found that among the 22 genes related to lipid metabolism and prognosis, CPNE3 was upregulated in epithelial cells (**Figures 12B, D**). To further analyze the changes in CPNE3 expression during tumor progression, we performed a pseudotime series analysis of epithelial cells. In the initial stage, the expression of CPNE3 gradually decreased over time (**Figure 12C**). These results contribute to our understanding of the single-cell population and variations in gene expression associated with lipid metabolism and prognosis at the cellular level (**Figure 12E**).

### 3.12 Immunohistochemical staining of key genes

**Figure 13:**
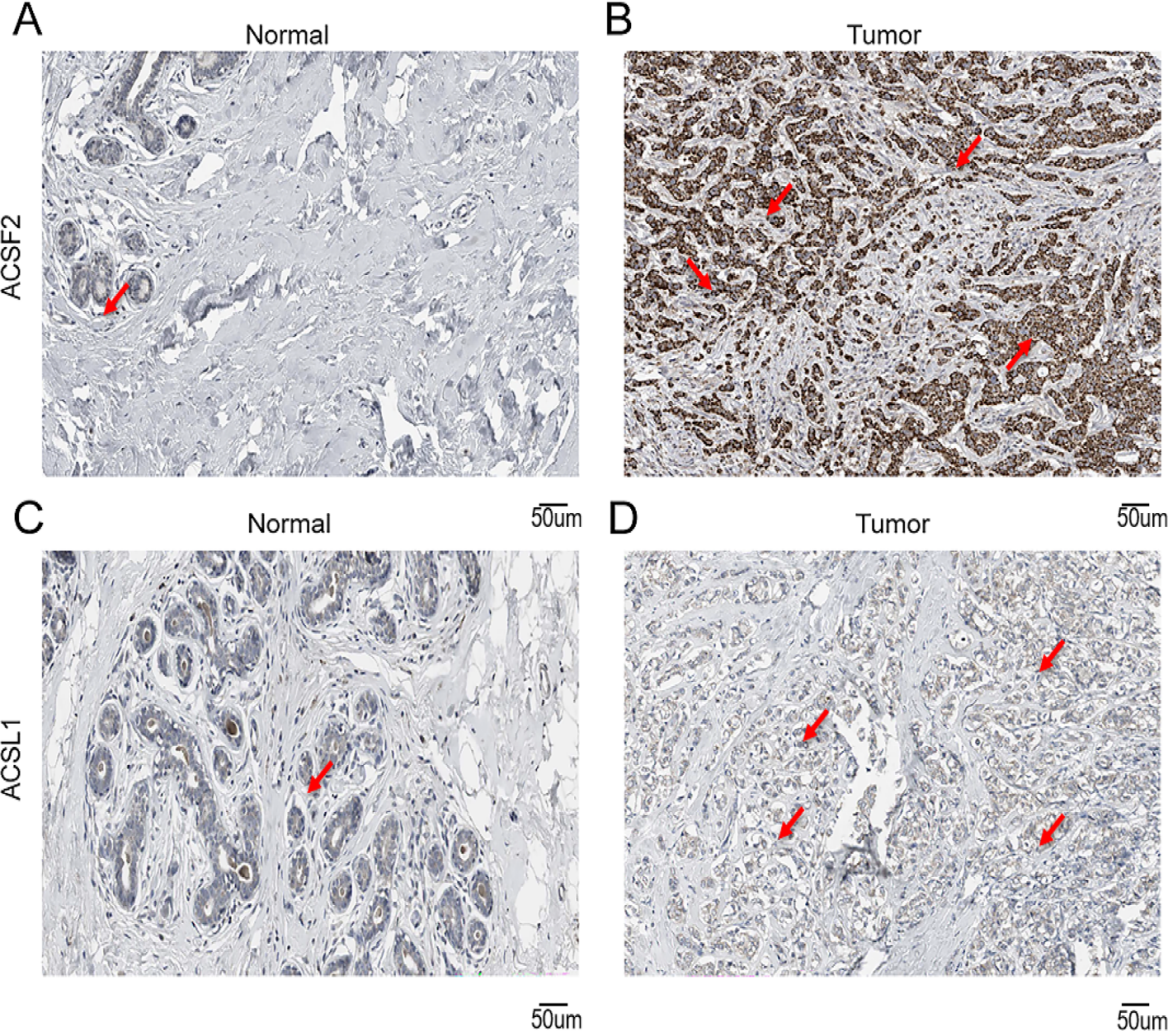
Immunohistochemical staining of key genes in human BRCA tumor tissues and normal tissues **(A)** IHC of ACSF2 in human normal tissues; **(B)** IHC of ACSF2 in human BRCA tissue samples; **(C)** IHC of ACSL1 in human normal tissue samples; **(D)** IHC of ACSL1 in human BRCA tissue samples.

To validate the disparities in the expression of crucial genes between human BRCA tissues and normal tissues, we obtained immunohistochemical profiles of key genes from the Human Protein Atlas database. We detected marked differences in the immunohistochemical staining of two key proteins, ACSF2 and ACSL1, between tumor tissue samples and normal tissue samples. As shown in the figure, the expression levels of ACSL1 and ACSF2 were higher in tumor tissues than in normal tissues (**Figures 13A–D**).

### 3.13 Verification of the expression and prognostic significance of hub genes

**Figure 14:**
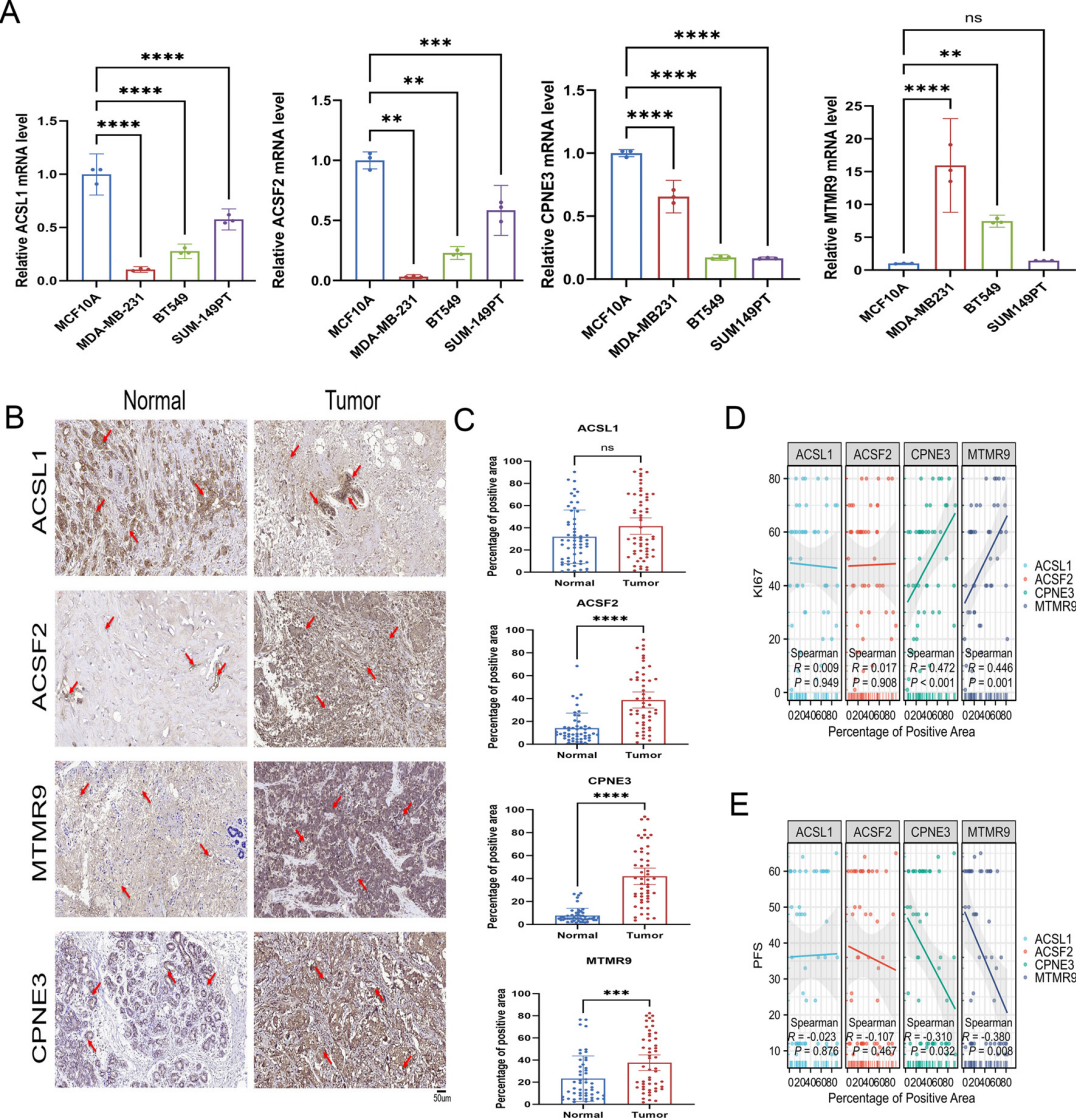
Verification of LMPGS hub genes expression. **(A)** PCR verification of the expression levels of the hub genes. **(B, C)** IHC was used to verify the expression of the hub genes in the cancer and adjacent tissues of 50 BRCA patients. **(D, E)** Correlation analysis of the hub gene expression levels with PFS and Ki67 in BRCA. (ns stands for p value>0.05; * stands for 0.01< p value <0.05; ** stands for 0.001< p value <0.01; *** stands for p value <0.001;**** stands for p value <0.0001).

According to the qRT−PCR results, the mRNA expression of MTMR9 and CPNE3 was greater in triple-negative breast cancer (TNBC) cell lines (MDA-MB-231 and BT549) and ER-, PR-, and HER2+ BRCA cell line (SUM149PT). However, ACSL1 and ACSF2 mRNA expression was greater in normal cell lines (nontumorigenic breast cell line: MCF10A) (**Figure 14A**). The MCF10A cell line is considered the baseline for evaluating changes in expression levels. IHC demonstrated the upregulation of ACSF2, MTMR9, and CPNE3 in BRCA tissues, whereas ACSL1 expression was not significantly different between tumor tissues and adjacent tissues (**Figures 14B, C**). Furthermore, a correlation analysis between the positive areas of each protein in tissue samples and progression-free survival (PFS) time was performed among 42 patients whose complete follow-up data were available. The results revealed that the expression levels of MTMR9 and CPNE3 were negatively correlated with the survival of BRCA patients. MTMR9, CPNE3 and Ki67 expression levels were positively correlated, and the difference was statistically significant (**Figures 14D, E**). However, no significant correlations were found between ACSL1 or ACSF2 expression and survival.

## 4 Discussion

Globally, BRCA is among the most prevalent cancers and ranks as the fifth leading cause of cancer-related mortality (33). Fatty acid metabolism profoundly influences the tumor immune microenvironment in BRCA, impacting disease progression and therapeutic responses (34–36). Despite advances in treatment modalities, a subset of patients with poor outcomes remains (37), highlighting the need for novel biomarkers to identify high-risk individuals. Therefore, developing accurate prognostic tools and increasing patient survival requires examining signals pertaining to fatty acid metabolism in BRCA prediction and therapy response. Omics approaches have become commonly utilized in cancer research as a means of identifying prognostic or diagnostic features and biomarkers (38). Specifically, omics-based risk stratification and molecular profiling could inform personalized treatment strategies. With advancements in medical technology and the evolution of data science, the application of machine learning in cancer research and treatment is garnering increasing attention and recognition (39). Moreover, the integration of machine learning with omics data is paving the way for new frontiers in cancer research. For instance, research has shown that using machine learning to analyze omics data can identify prognostic biomarkers for colorectal cancer (40). Additionally, in TNBC, omics analysis revealed distinct metabolic subtypes with varying responses to specific inhibitors, suggesting potential targeted therapeutic approaches (41). However, the application of machine learning combined with omics analysis to determine the molecular characteristics of BRCA remains limited. Our study aimed to develop a novel omics-based model for patient prognostication and risk stratification in BRCA patients that could advance personalized treatment. Additionally, our goal was to clarify the molecular processes that underlie the correlation between BRCA prognosis and treatment response.

To determine the important genes about fatty acid metabolism, a cluster analysis on 1280 BRCA samples was conducted in this research. GSVA revealed a strong correlation between fatty acid metabolism and the IL6-STAT3, PI3K-AKT-MTOR, E2F TARGETS, and MYC TARGETS pathways, demonstrating the strong connection between fatty acid metabolism and biological processes related to BRCA. By employing nine machine learning techniques and exploring their 184 combinations to screen feature variables, an optimal prognostic model consisting of 21 genes was developed. The algorithms we selected, such as LASSO, Ridge, and Elastic Net, are excellent at handling high-dimensional data and multicollinearity, and have wide applications in survival analysis and prognostic modeling. By combining different types of machine learning algorithms, we can improve the robustness and accuracy of the model to capture the diverse interpretations of the data by each algorithm. The LMPGS model may successfully stratify BRCA patients according to risk and OS and may function as an independent prognostic factor, as shown by KM curve analysis and multivariate analysis. Furthermore, the GSE88770 and GSE20711 datasets were utilized as validation datasets to confirm their efficacy. These findings highlight the excellent predictive ability of the proposed LMPGS model for assessing prognosis in BRCA patients; notably, the model also has applicability across various other cancers. Our results show that while the validation set confirms the model’s effectiveness, its poor performance is still a significant concern. Although Elastic Net combined with LASSO performed best on the training set, it may overfit the specific noise and features of that set, leading to weak generalization on new data. As a result, the model’s performance on the validation set is unsatisfactory. Additionally, variations in datasets—such as differences in patient populations, disease subtypes, or data collection methods—could impact the model, which includes 21 prognostic genes with varying effects across datasets. To address these issues, we plan to expand our data and improve the model’s generalizability and prediction accuracy through more rigorous validation and tuning. Moreover, we found a high LMPGS score was correlated with advanced stages of BRCA and higher tumor grades. Our results illustrated that tumors in the high-score group exhibited higher invasiveness. Therefore, this scoring system serves as a valuable tool for the early identification of high-risk patients. By accurately predicting disease progression, clinicians can implement more targeted early interventions, potentially improving patient survival. To facilitate the prediction of BRCA prognosis in individual patients, we created a nomogram that combines clinical features with the LMPGS score. The C-index and ROC curve showed that the nomogram had good discriminatory capacity, indicating high predictive accuracy. Crucially, compared with other clinical features, our nomogram exhibited superior net benefit when applied in predicting survival outcomes for BRCA patients, demonstrating its potential as a useful and promising therapeutic tool.

This systematic exploration of the omics differences in low-grade mucinous BRCA patients stratified by risk according to the LMPGS model provides a comprehensive understanding of the underlying regulatory mechanisms involved. Intratumoral heterogeneity (ITH), characterized by the accumulation of gene mutations (42), is a well-known genetic characteristic of cancer that has been linked to malignancy and heightened treatment resistance (43). In the high-LMPGS score group, significant amplification was observed at 8q24.21, while significant deletion occurred at 8p21.3. Previous studies have demonstrated that alterations in chromosome 8 are closely linked to BRCA prognosis and treatment response (44) and serve as indicators of a poor prognosis (45); thus, these alterations might contribute to unfavorable outcomes in patients in the high-score group, which is consistent with our findings. Furthermore, MYC is a powerful oncogene found at 8q24.21 (46), which provides more evidence for the connection between the MYC pathway and fatty acid metabolism. As an epigenetic modification, DNA methylation is essential for controlling gene transcription and preserving the integrity of the genome (47). Research uncovered that the methylation levels of ACSL1 and ACSF2 were notably higher in the low-score group, so methylation of these genes may contribute to the observed differences in prognosis among the two groups.

In the last decade, ICI have emerged as crucial therapeutic agents for solid tumors (48, 49). Our findings demonstrated significant upregulation of immune checkpoint molecules, including CD40 and TNFRSF9, in patients with low LMPGS scores, whereas patients with high LMPGS scores presented higher levels of CD80 expression. Furthermore, the TIDE algorithm is a widely acknowledged tool for predicting the responsiveness of patients with tumors to ICI based on their expression profiles (50). TIDE score analysis predicted a greater immunotherapy response rate in patients with high LMPGS scores and indicated that ICI efficacy may be compromised and the immunological escape potential may be greater in the low-score group. The TMB indirectly indicates the tumor’s ability and extent of neoantigen production, thus predicting the effectiveness of immunotherapy across various cancers (51). The higher TMB observed in the high-rated group suggests a potentially more favorable response to immunotherapy, reinforcing our analysis. In the low-rated group, genes associated with inflammation and immune response, such as IFNAR2, CXCL10, and TLR1 (52–54), showed heightened expression, promoting increased immune cell infiltration. Our analysis revealed that this group exhibited a higher immune score. However, there were also elevated levels of immunosuppressive molecules such as PDCD1 and IDO1 (55, 56), potentially impairing immune cell function. This dual pattern may explain the poor response to immunotherapy observed in these cases. These findings indicate that while increased immune cell presence is observed, it does not necessarily correlate with heightened sensitivity to immunotherapy. The presence of intricate immune microenvironments and immunosuppressive mechanisms likely plays a crucial role in influencing treatment outcomes. Furthermore, drug validation experiments confirmed that the high-score group had superior efficacy of drug therapy. These results imply that our LMPGS model could be useful for the early identification of BRCA patients who will likely benefit from first-line immunotherapy. Although this study revealed some discoveries in the exploration of the relationships between the LMPGS group and immune-related genes, several limitations remain. First, our analysis focused on the correlation of gene expression levels, and did not delve into the complex regulatory mechanisms of genes at the transcriptional level, and did not conduct coexpression network analysis, transcription factor analysis, or epigenetic modification assessment. As a result, we are unable to fully understand the complex interactions and regulatory relationships between these genes. In addition, limitations in sample size and data sources may affect the generalizability of results. To overcome these limitations, future studies should combine multilevel bioinformatic analyses and functional experiments to fully understand the interactions and regulatory mechanisms between LMPGS genes and immune-related genes. These findings will provide a more reliable scientific basis for clinical application.

Early identification of treatment-sensitive patients by clinicians is necessary for personalized treatment. To further identify potentially effective drugs, we integrated the CCLE and PRISM databases and identified seven potential drugs. Molecular docking technology was used to identify niclosamide as a drug candidate that interacts with key genes associated with LMPGS and inhibits STAT3 activation (57), which is aberrantly activated in BRCA. STAT3 activation promotes angiogenesis, tumor invasion, metastasis, and cell cycle progression. Previous studies have shown that niclosamide can prevent adipocytes from undergoing epithelial−mesenchymal transition via the paracrine IL-6/Stat3 signaling pathway, thereby suppressing the occurrence and development of BRCA (58). Molecular docking analysis provides preliminary clues that niclosamide can bind to the key lipid metabolism prognostic genes ACSF2 and MTMR9, but there are significant limitations. These limitations include limited model training data, possible simulation errors, and a lack of experimental data support. Our preliminary findings therefore require more experimental validation to ensure their biological relevance. In addition, molecular docking models focus mainly on possible direct binding sites, but the actual action of drugs in the cell may involve more complex mechanisms, including indirect regulatory effects and systemic effects, and these complexities are often not fully reflected in docking models. For example, new binding sites interact with known drug targets, and lipid genes influence drug metabolism to achieve their biological effects, details that have not been explored in depth via molecular docking. Although we observed the antitumor effects of niclosamide on BRCA cells and its effect on MTMR9 expression levels, these experimental results are still preliminary and have not been systematically replicated and validated. Therefore, in order to fully understand the mechanism of action of the drug, further validation *in vivo* models and clinical samples is needed and future studies should focus on verifying the biological relevance of these predicted results and examining how the drug affects lipid metabolism and other biological processes through more complex mechanisms. Additional clinical studies are required to validate the extensive therapeutic potential of niclosamide in BRCA treatment.

However, further validation is warranted. After model selection and careful study, key prognostic genes were identified. ACSL1, an essential rate-limiting enzyme in lipid metabolism, catalyzes the synthesis of phospholipids, cholesterol esters, triglycerides, and energy-producing fatty acids (59) and participates in the formation of lipid droplets. Low ACSL1 expression is associated with a better prognosis in patients with IDH1-mutant glioma (60). ACSL1 mediates ferroptosis and inhibits tumor growth in TNBC (61). However, in MDA-231 cells, ACSL1 plays a crucial role in regulating the excessive production of TNFα-mediated inflammatory processes related to tumor growth. ACSL1 promotes the progression of ovarian cancer by regulating FSP1 myristoylation to increase antioxidant capacity and ferroptosis resistance (62). Therefore, the current role of ACSL1 in cancer is still controversial. In the present study, the expression of ACSL1 in cell lines derived from TNBC, which has a worse prognosis, was lower than that in non-TNBC cell lines, which was consistent with the findings of the bioinformatics analysis in an earlier paper. However, no significant correlation between clinical specimen validation and prognosis was observed in this study and further mechanistic study is needed. It is worth noting that the immunohistochemical staining results of ACSL1 in HPA database and clinical patients are different, and the different conclusions of ACSL1 may be related to the specific characteristics of the samples or experimental conditions and further mechanistic studies are needed. Notably, the results of the immunohistochemical staining of ACSL1 in the HPA database differ from those in clinical patients, and the different conclusions regarding ACSL1 may be related to the specific characteristics of the samples or experimental conditions. This difference may be due to differences in posttranscriptional regulation and translation efficiency. In addition, the heterogeneity of sample sources may also lead to differences in experimental results. ACSF2,an enzyme acyl-CoA synthetase family member 2, controls the oxidation of fatty acids and lipid metabolism. The expression of ACSF2 increases synergistically after etoposide treatment, confirming that the treatment induces ferroptosis in ER-positive BRCA cells (63). Consistent with previous studies, this finding further verified the inhibitory effect of ACSF2 on tumors, but further functional experiments are still needed for verification. MTMR9 belongs to the myosin-related protein family and is mainly a bispecific phosphatase. Some studies have shown that it is identified as lipid phosphatase (64) MTMR9, which lacks a phosphatase domain, has been demonstrated to improve the functionality of other MTMR proteins, including MTMR6 and MTMR7 (65, 66). Research has indicated a correlation between high MTMR9 expression and poor outcomes in patients with esophageal cancer (67). However, no association between MTMR9 and BRCA has been reported. This study revealed, for the first time, that MTMR9, a gene involved in fatty acid metabolism, is correlated with poor prognosis in BRCA patients. At the single-cell level, we identified eight distinct cell subgroups, with CPNE3 exhibiting high expression in epithelial cells. Previous studies have demonstrated that CPNE3 triggers the PI3K/AKT signaling pathway to regulate the proliferation and apoptosis of human glioblastoma cells (68). Moreover, CPNE3 overexpression significantly increases the metastatic potential of BRCA cells (69). Furthermore, single-cell pseudotime analysis revealed robust upregulation of CPNE3 in early-stage BRCA, suggesting its potential utility as an early diagnostic marker. In addition, CPNE3 was highly expressed in IHC analyses of BRCA patients and was significantly associated with prognosis. The key genes we identified, MTMR9 and CPNE3, were significantly different between patients with high and low lipid metabolism scores, suggesting that they may play an important roles in the biological behavior of BRCA. While our study highlights the relevance of these genes to BRCA prognosis, direct functional experiments to verify their biological role in tumor progression are currently lacking. Future studies should focus on conducting *in vitro* and *in vivo* experiments to clarify the specific mechanisms of MTMR9 and CPNE3 in cell proliferation, migration, apoptosis and other biological processes. For example, the role of these two genes in BRCA cell lines can be evaluated through gene knockout or overexpression experiments, combined with cell biology techniques. In addition, our analysis shows that some mutation information associated with these two genes can be found from public databases such as the TCGA. The presence of these mutations may be related to tumor biological characteristics, prognostic manifestations and treatment outcomes, but more research is needed to support these aspects. For example, the effects of different mutation types on MTMR9 and CPNE3 expression levels can be analyzed to understand how these mutations drive disease progression in the tumor microenvironment. Moreover, niclosamide can inhibit tumor proliferation and affect the expression of the target gene MTMR9, which further reflects the correlation between MTMR9 and the occurrence and development of BRCA. In conclusion, future studies should focus on functional validation and mutation analysis of MTMR9 and CPNE3 to understand their biological significance in the development of BRCA. These findings are expected to provide new biomarkers and targets for the development of personalized treatment strategies.

To mitigate the potential influence of personal preferences on modeling methods, we employed a comprehensive approach by combining nine well-established machine learning algorithms into 184 combinations and selecting the optimal model on the basis of its accuracy. Despite our rigorous efforts and promising results, it is critical to recognize some of our study’s shortcomings. Research has demonstrated that tumor heterogeneity can impact the efficacy of immunotherapy or chemotherapy. A significant limitation concerns the potential for intratumor or intrapatient tumor heterogeneity. The study datasets are publicly available high-throughput sequencing datasets based on different platforms and are prone to batch effects. Although we have extensively assessed and validated the LMPGS signatures, the limited sample size, incomplete data, and insufficient clinical validation limit the applicability of our findings. Thus, to further validate our findings, extensive multicenter prospective investigations are necessary. Finally, although the sensitivity of patients in different LMPGS risk categories to different small-molecule medicines is anticipated, confirming this finding via *in vitro* pharmacokinetic research and clinical trials is crucial.

## 5 Conclusion

Our research demonstrated the LMPGS model’s correlation with immune infiltration features and immunotherapy response, underscoring its potential as a valid prognostic indicator for BRCA. In BRCA patients, the LMPGS model is a useful tool for prognostication and therapeutic decision making. It can also be used to identify patients who may benefit from chemotherapy or anticancer immunotherapy. Our thorough examination of fatty acid metabolism-related genes provides important new information about their possible significance and function in BRCA. Through *in vitro* experiments and clinical data validation, MTMR9 and CPNE3 were screened as key markers for BRCA prognosis, and niclosamide was identified as a potential target drug. Overall, our findings enhance the understanding of lipid metabolic reprogramming in BRCA and provide an attractive approach for prognostic assessment, risk stratification, and personalized treatment of patients with BRCA in clinical practice.

## Data Availability

The datasets presented in this study can be found in online repositories. The names of the repository/repositories and accession number(s) can be found in the article/**Supplementary Material**.

## References

[1] Schick J Ritchie RP Restini C. Breast cancer therapeutics and biomarkers: past, present, and future approaches. Breast Cancer (Auckl). (2021) 15:1178223421995854. doi: 10.1177/1178223421995854 33994789 PMC8100889

[2] Harbeck N Penault-Llorca F Cortes J Gnant M Houssami N Poortmans P. Breast cancer. Nat Rev Dis Primers. (2019) 5:66. doi: 10.1038/s41572-019-0111-2 31548545

[3] Stickeler E. Prognostic and predictive markers for treatment decisions in early breast cancer. Breast Care (Basel). (2011) 6:193–8. doi: 10.1159/000329471 PMC3132966 21779224

[4] Leon-Ferre RA Goetz MP. Advances in systemic therapies for triple negative breast cancer. Bmj. (2023) 381:e071674. doi: 10.1136/bmj-2022-071674 37253507

[5] Meattini I Livi L Lorito N Becherini C Bacci M Visani L. Integrating radiation therapy with targeted treatments for breast cancer: From bench to bedside. Cancer Treat Rev. (2022) 108:102417. doi: 10.1016/j.ctrv.2022.102417 35623219

[6] Trayes KP Cokenakes SEH. Breast cancer treatment. Am Fam Physician. (2021) 104:171–8. 34383430

[7] Taneja P Maglic D Kai F Zhu S Kendig RD Fry EA. Classical and novel prognostic markers for breast cancer and their clinical significance. Clin Med Insights Oncol. (2010) 4:15–34. doi: 10.4137/CMO.S4773 20567632 PMC2883240

[8] Wu HJ Chu PY. Recent discoveries of macromolecule- and cell-based biomarkers and therapeutic implications in breast cancer. Int J Mol Sci. (2021) 22:636. doi: 10.3390/ijms22020636 33435254 PMC7827149

[9] Pitt JM Marabelle A Eggermont A Soria JC Kroemer G Zitvogel L. Targeting the tumor microenvironment: removing obstruction to anticancer immune responses and immunotherapy. Ann Oncol. (2016) 27:1482–92. doi: 10.1093/annonc/mdw168 27069014

[10] DeBerardinis RJ Chandel NS. Fundamentals of cancer metabolism. Sci Adv. (2016) 2:e1600200. doi: 10.1126/sciadv.1600200 27386546 PMC4928883

[11] Pham DV Park PH. Adiponectin triggers breast cancer cell death via fatty acid metabolic reprogramming. J Exp Clin Cancer Res. (2022) 41:9. doi: 10.1186/s13046-021-02223-y 34986886 PMC8729140

[12] Snaebjornsson MT Janaki-Raman S Schulze A. Greasing the wheels of the cancer machine: the role of lipid metabolism in cancer. Cell Metab. (2020) 31:62–76. doi: 10.1016/j.cmet.2019.11.010 31813823

[13] Xia X Huang C Liao Y Liu Y He J Shao Z. The deubiquitinating enzyme USP15 stabilizes ERα and promotes breast cancer progression. Cell Death Dis. (2021) 12:329. doi: 10.1038/s41419-021-03607-w 33771975 PMC7997968

[14] Zipinotti Dos Santos D de Souza JC Pimenta TM da Silva Martins B Junior RSR Butzene SMS. The impact of lipid metabolism on breast cancer: a review about its role in tumorigenesis and immune escape. Cell Commun Signal. (2023) 21:161. doi: 10.1186/s12964-023-01178-1 37370164 PMC10304265

[15] Wang T Fahrmann JF Lee H Li YJ Tripathi SC Yue C. JAK/STAT3-regulated fatty acid β-oxidation is critical for breast cancer stem cell self-renewal and chemoresistance. Cell Metab. (2018) 27:136–50.e5. doi: 10.1016/j.cmet.2017.11.001 29249690 PMC5777338

[16] Xiao Q Xia M Tang W Zhao H Chen Y Zhong J. The lipid metabolism remodeling: A hurdle in breast cancer therapy. Cancer Lett. (2024) 582:216512. doi: 10.1016/j.canlet.2023.216512 38036043

[17] Goldman MJ Craft B Hastie M Repečka K McDade F Kamath A. Visualizing and interpreting cancer genomics data via the Xena platform. Nat Biotechnol. (2020) 38:675–8. doi: 10.1038/s41587-020-0546-8 PMC7386072 32444850

[18] Cerami E Gao J Dogrusoz U Gross BE Sumer SO Aksoy BA. The cBio cancer genomics portal: an open platform for exploring multidimensional cancer genomics data. Cancer Discovery. (2012) 2:401–4. doi: 10.1158/2159-8290.CD-12-0095 PMC3956037 22588877

[19] Metzger-Filho O Michiels S Bertucci F Catteau A Salgado R Galant C. Genomic grade adds prognostic value in invasive lobular carcinoma. Ann Oncol. (2013) 24:377–84. doi: 10.1093/annonc/mds280 23028037

[20] Dedeurwaerder S Desmedt C Calonne E Singhal SK Haibe-Kains B DeFrance M. DNA methylation profiling reveals a predominant immune component in breast cancers. EMBO Mol Med. (2011) 3:726–41. doi: 10.1002/emmm.201100801 PMC3377115 21910250

[21] Clough E Barrett T. The gene expression omnibus database. Methods Mol Biol. (2016) 1418:93–110. doi: 10.1007/978-1-4939-3578-9_5 27008011 PMC4944384

[22] Xu K Wang R Xie H Hu L Wang C Xu J. Single-cell RNA sequencing reveals cell heterogeneity and transcriptome profile of breast cancer lymph node metastasis. Oncogenesis. (2021) 10:66. doi: 10.1038/s41389-021-00355-6 34611125 PMC8492772

[23] Liberzon A Subramanian A Pinchback R Thorvaldsdóttir H Tamayo P Mesirov JP. Molecular signatures database (MSigDB) 3.0. Bioinformatics. (2011) 27:1739–40. doi: 10.1093/bioinformatics/btr260 PMC3106198 21546393

[24] Li C Tao Y Chen Y Wu Y He Y Yin S. Development of a metabolism-related signature for predicting prognosis, immune infiltration and immunotherapy response in breast cancer. Am J Cancer Res. (2022) 12:5440–61. PMC9827085 36628282

[25] Li W Gao Y Jin X Wang H Lan T Wei M. Comprehensive analysis of N6-methylandenosine regulators and m6A-related RNAs as prognosis factors in colorectal cancer. Mol Ther Nucleic Acids. (2022) 27:598–610. doi: 10.1016/j.omtn.2021.12.007 35070494 PMC8753275

[26] Liu Y Wang J Jiang M. Copper-related genes predict prognosis and characteristics of breast cancer. Front Immunol. (2023) 14:1145080. doi: 10.3389/fimmu.2023.1145080 37180167 PMC10172490

[27] Luo W Han Y Li X Liu Z Meng P Wang Y. Breast cancer prognosis prediction and immune pathway molecular analysis based on mitochondria-related genes. Genet Res (Camb). (2022) 2022:2249909. doi: 10.1155/2022/2249909 35707265 PMC9174003

[28] Zhou Y Zheng J Bai M Gao Y Lin N. Effect of pyroptosis-related genes on the prognosis of breast cancer. Front Oncol. (2022) 12:948169. doi: 10.3389/fonc.2022.948169 35957895 PMC9357945

[29] Subramanian A Tamayo P Mootha VK Mukherjee S Ebert BL Gillette MA. Gene set enrichment analysis: a knowledge-based approach for interpreting genome-wide expression profiles. Proc Natl Acad Sci U.S.A. (2005) 102:15545–50. doi: 10.1073/pnas.0506580102 PMC1239896 16199517

[30] Fu J Li K Zhang W Wan C Zhang J Jiang P. Large-scale public data reuse to model immunotherapy response and resistance. Genome Med. (2020) 12:21. doi: 10.1186/s13073-020-0721-z 32102694 PMC7045518

[31] Nusinow DP Szpyt J Ghandi M Rose CM McDonald ER 3rd Kalocsay M. Quantitative proteomics of the cancer cell line encyclopedia. Cell. (2020) 180:387–402.e16. doi: 10.1016/j.cell.2019.12.023 31978347 PMC7339254

[32] Uhlén M Fagerberg L Hallström BM Lindskog C Oksvold P Mardinoglu A. Proteomics. Tissue-based map of the human proteome. Science. (2015) 347:1260419. doi: 10.1126/science.1260419 25613900

[33] Sung H Ferlay J Siegel RL Laversanne M Soerjomataram I Jemal A. Global cancer statistics 2020: GLOBOCAN estimates of incidence and mortality worldwide for 36 cancers in 185 countries. CA Cancer J Clin. (2021) 71:209–49. doi: 10.3322/caac.21660 33538338

[34] Cheng C Geng F Cheng X Guo D. Lipid metabolism reprogramming and its potential targets in cancer. Cancer Commun (Lond). (2018) 38:27. doi: 10.1186/s40880-018-0301-4 29784041 PMC5993136

[35] Sun H Zhang L Wang Z Gu D Zhu M Cai Y. Single-cell transcriptome analysis indicates fatty acid metabolism-mediated metastasis and immunosuppression in male breast cancer. Nat Commun. (2023) 14:5590. doi: 10.1038/s41467-023-41318-2 37696831 PMC10495415

[36] Yu W Lei Q Yang L Qin G Liu S Wang D. Contradictory roles of lipid metabolism in immune response within the tumor microenvironment. J Hematol Oncol. (2021) 14:187. doi: 10.1186/s13045-021-01200-4 34742349 PMC8572421

[37] Ye F Dewanjee S Li Y Jha NK Chen ZS Kumar A. Advancements in clinical aspects of targeted therapy and immunotherapy in breast cancer. Mol Cancer. (2023) 22:105. doi: 10.1186/s12943-023-01805-y 37415164 PMC10324146

[38] He X Liu X Zuo F Shi H Jing J. Artificial intelligence-based multi-omics analysis fuels cancer precision medicine. Semin Cancer Biol. (2023) 88:187–200. doi: 10.1016/j.semcancer.2022.12.009 36596352

[39] Sarkar C Das B Rawat VS Wahlang JB Nongpiur A Tiewsoh I. Artificial intelligence and machine learning technology driven modern drug discovery and development. Int J Mol Sci. (2023) 24:2026. doi: 10.3390/ijms24032026 36768346 PMC9916967

[40] Wei W Li Y Huang T. Using machine learning methods to study colorectal cancer tumor micro-environment and its biomarkers. Int J Mol Sci. (2023) 24:11133. doi: 10.3390/ijms241311133 37446311 PMC10342679

[41] Gong Y Ji P Yang YS Xie S Yu TJ Xiao Y. Metabolic-pathway-based subtyping of triple-negative breast cancer reveals potential therapeutic targets. Cell Metab. (2021) 33:51–64.e9. doi: 10.1016/j.cmet.2020.10.012 33181091

[42] Samstein RM Lee CH Shoushtari AN Hellmann MD Shen R Janjigian YY. Tumor mutational load predicts survival after immunotherapy across multiple cancer types. Nat Genet. (2019) 51:202–6. doi: 10.1038/s41588-018-0312-8 PMC6365097 30643254

[43] Dentro SC Leshchiner I Haase K Tarabichi M Wintersinger J Deshwar AG. Characterizing genetic intra-tumor heterogeneity across 2,658 human cancer genomes. Cell. (2021) 184:2239–54.e39. doi: 10.1016/j.cell.2021.03.009 33831375 PMC8054914

[44] Han S Park K Shin E Kim HJ Kim JY Kim JY. Genomic change of chromosome 8 predicts the response to taxane-based neoadjuvant chemotherapy in node-positive breast cancer. Oncol Rep. (2010) 24:121–8. 20514452

[45] Horlings HM Lai C Nuyten DS Halfwerk H Kristel P van Beers E. Integration of DNA copy number alterations and prognostic gene expression signatures in breast cancer patients. Clin Cancer Res. (2010) 16:651–63. doi: 10.1158/1078-0432.CCR-09-0709 20068109

[46] Cai Q Medeiros LJ Xu X Young KH. MYC-driven aggressive B-cell lymphomas: biology, entity, differential diagnosis and clinical management. Oncotarget. (2015) 6:38591–616. doi: 10.18632/oncotarget.v6i36 PMC4770723 26416427

[47] Papanicolau-Sengos A Aldape K. DNA methylation profiling: an emerging paradigm for cancer diagnosis. Annu Rev Pathol. (2022) 17:295–321. doi: 10.1146/annurev-pathol-042220-022304 34736341

[48] Haanen J Ernstoff MS Wang Y Menzies AM Puzanov I Grivas P. Autoimmune diseases and immune-checkpoint inhibitors for cancer therapy: review of the literature and personalized risk-based prevention strategy. Ann Oncol. (2020) 31:724–44. doi: 10.1016/j.annonc.2020.03.285 32194150

[49] Billan S Kaidar-Person O Gil Z. Treatment after progression in the era of immunotherapy. Lancet Oncol. (2020) 21:e463–e76. doi: 10.1016/S1470-2045(20)30328-4 33002442

[50] Jiang P Gu S Pan D Fu J Sahu A Hu X. Signatures of T cell dysfunction and exclusion predict cancer immunotherapy response. Nat Med. (2018) 24:1550–8. doi: 10.1038/s41591-018-0136-1 PMC6487502 30127393

[51] Yarchoan M Hopkins A Jaffee EM. Tumor mutational burden and response rate to PD-1 inhibition. N Engl J Med. (2017) 377:2500–1. doi: 10.1056/NEJMc1713444 PMC6549688 29262275

[52] Jia DJ Wang QW Hu YY He JM Ge QW Qi YD. Lactobacillus johnsonii alleviates colitis by TLR1/2-STAT3 mediated CD206(+) macrophages(IL-10) activation. Gut Microbes. (2022) 14:2145843. doi: 10.1080/19490976.2022.2145843 36398889 PMC9677986

[53] Lamrani M Sassi N Paul C Yousfi N Boucher JL Gauthier N. TLR4/IFNγ pathways induce tumor regression via NOS II-dependent NO and ROS production in murine breast cancer models. Oncoimmunology. (2016) 5:e1123369. doi: 10.1080/2162402X.2015.1123369 27467924 PMC4910700

[54] Tokunaga R Zhang W Naseem M Puccini A Berger MD Soni S. CXCL9, CXCL10, CXCL11/CXCR3 axis for immune activation - A target for novel cancer therapy. Cancer Treat Rev. (2018) 63:40–7. doi: 10.1016/j.ctrv.2017.11.007 PMC5801162 29207310

[55] Ribas A. Tumor immunotherapy directed at PD-1. N Engl J Med. (2012) 366:2517–9. doi: 10.1056/NEJMe1205943 22658126

[56] Zhai L Ladomersky E Lenzen A Nguyen B Patel R Lauing KL. IDO1 in cancer: a Gemini of immune checkpoints. Cell Mol Immunol. (2018) 15:447–57. doi: 10.1038/cmi.2017.143 PMC6068130 29375124

[57] Wu MM Zhang Z Tong CWS Yan VW Cho WCS To KKW. Repurposing of niclosamide as a STAT3 inhibitor to enhance the anticancer effect of chemotherapeutic drugs in treating colorectal cancer. Life Sci. (2020) 262:118522. doi: 10.1016/j.lfs.2020.118522 33011217

[58] Gyamfi J Lee YH Min BS Choi J. Niclosamide reverses adipocyte induced epithelial-mesenchymal transition in breast cancer cells via suppression of the interleukin-6/STAT3 signalling axis. Sci Rep. (2019) 9:11336. doi: 10.1038/s41598-019-47707-2 31383893 PMC6683291

[59] Li LO Ellis JM Paich HA Wang S Gong N Altshuller G. Liver-specific loss of long chain acyl-CoA synthetase-1 decreases triacylglycerol synthesis and beta-oxidation and alters phospholipid fatty acid composition. J Biol Chem. (2009) 284:27816–26. doi: 10.1074/jbc.M109.022467 PMC2788832 19648649

[60] Zhou L Wang Z Hu C Zhang C Kovatcheva-Datchary P Yu D. Integrated metabolomics and lipidomics analyses reveal metabolic reprogramming in human glioma with IDH1 mutation. J Proteome Res. (2019) 18:960–9. doi: 10.1021/acs.jproteome.8b00663 30596429

[61] Beatty A Singh T Tyurina YY Tyurin VA Samovich S Nicolas E. Ferroptotic cell death triggered by conjugated linolenic acids is mediated by ACSL1. Nat Commun. (2021) 12:2244. doi: 10.1038/s41467-021-22471-y 33854057 PMC8046803

[62] Zhang Q Li N Deng L Jiang X Zhang Y Lee LTO. ACSL1-induced ferroptosis and platinum resistance in ovarian cancer by increasing FSP1 N-myristylation and stability. Cell Death Discovery. (2023) 9:83. doi: 10.1038/s41420-023-01385-2 36882396 PMC9992462

[63] Ozkan E Bakar-Ates F. Etoposide in combination with erastin synergistically altered iron homeostasis and induced ferroptotic cell death through regulating IREB2/FPN1 expression in estrogen receptor positive-breast cancer cells. Life Sci. (2023) 312:121222. doi: 10.1016/j.lfs.2022.121222 36442526

[64] Guo L Martens C Bruno D Porcella SF Yamane H Caucheteux SM. Lipid phosphatases identified by screening a mouse phosphatase shRNA library regulate T-cell differentiation and protein kinase B AKT signaling. Proc Natl Acad Sci U.S.A. (2013) 110:E1849–56. doi: 10.1073/pnas.1305070110 PMC3657794 23630283

[65] Mochizuki Y Majerus PW. Characterization of myotubularin-related protein 7 and its binding partner, myotubularin-related protein 9. Proc Natl Acad Sci U.S.A. (2003) 100:9768–73. doi: 10.1073/pnas.1333958100 PMC187840 12890864

[66] Zou J Chang SC Marjanovic J Majerus PW. MTMR9 increases MTMR6 enzyme activity, stability, and role in apoptosis. J Biol Chem. (2009) 284:2064–71. doi: 10.1074/jbc.M804292200 PMC2629094 19038970

[67] Goh XY Rees JR Paterson AL Chin SF Marioni JC Save V. Integrative analysis of array-comparative genomic hybridisation and matched gene expression profiling data reveals novel genes with prognostic significance in oesophageal adenocarcinoma. Gut. (2011) 60:1317–26. doi: 10.1136/gut.2010.234179 21478220

[68] Zhang D Wang X Wang X Wang Z Ma S Zhang C. CPNE3 regulates the cell proliferation and apoptosis in human Glioblastoma via the activation of PI3K/AKT signaling pathway. J Cancer. (2021) 12:7277–86. doi: 10.7150/jca.60049 PMC8734413 35003348

[69] Heinrich C Keller C Boulay A Vecchi M Bianchi M Sack R. Copine-III interacts with ErbB2 and promotes tumor cell migration. Oncogene. (2010) 29:1598–610. doi: 10.1038/onc.2009.456 20010870

