# Supplementary material for: Integration of transcriptomics and machine learning for insights into breast cancer: exploring lipid metabolism and immune interactions
Source: Front Immunol. 2024 Oct 25;15:1470167. doi: 10.3389/fimmu.2024.1470167 (PMC11543460; doi:10.3389/fimmu.2024.1470167)
Supplement: Supplementary file 1 [file DataSheet1.docx]

Supplementary Material

# Supplementary Table

**Supplementary Table S1**. Primers sequences were shown.

| **Supplementary Table S1. Primers for qRT-PCR detection** | | |
| --- | --- | --- |
| ACSL1 | Forward | CTCTTCCGACCAACACGCTTATG |
|  | Reverse | CACCACTACCCGCCACTTCC |
| ACSF2 | Forward | CTCTTCCGACCAACACGCTTATG |
|  | Reverse | CACCACTACCCGCCACTTCC |
| MTMR9 | Forward | CATAACATGGACCGATGGCTCAG |
|  | Reverse | GGCAGGCAGTTGTCAGAATCTC |
| CPNE3 | Forward | CAGCCACGCAACAGCAGAC |
|  | Reverse | AGCACCTCCAACTCCAACAATTATG |
| AKT | Forward | ATGAACGACGTAGCCATTGTG |
|  | Reverse | TTGTAGCCAATAAAGGTGCCAT |
| PI3K | Forward | TGTGGAGCTCGCTAAAGTCA |
|  | Reverse | CACTCCTGCCCTAAATGGGA |
| CCND1 | Forward | TGGATGCTGGAGGTCTGTGAGG |
|  | Reverse | TTGCGGATGGTCTGCTTGTTCTC |
| BCL-2 | Forward | GCGGCCTCTGTTTGATTTCT |
|  | Reverse | TCACTTGTGGCCCAGATAGG |
| TUBULIN | Forward | GCGCTTATCGAAGTGTGGTC |
|  | Reverse | ACCCTTCCCCTAGACACTCG |

# Supplementary Figures

## Supplementary FigureS1


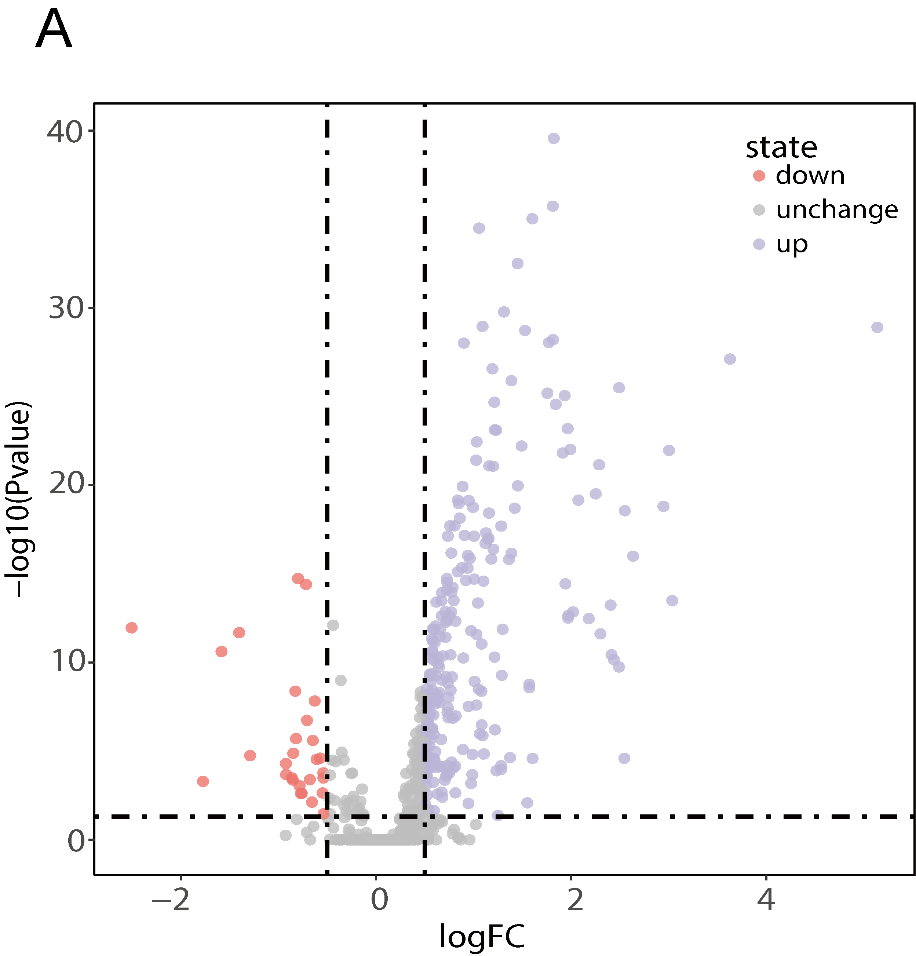


**Supplementary Figure S1.** **Screening of differentially expressed genes between high and low lipid metabolism score groups**

Volcano plot of differentially expressed genes between high and low lipid metabolism score groups in the TCGA-BRCA training set.

## Supplementary FigureS2


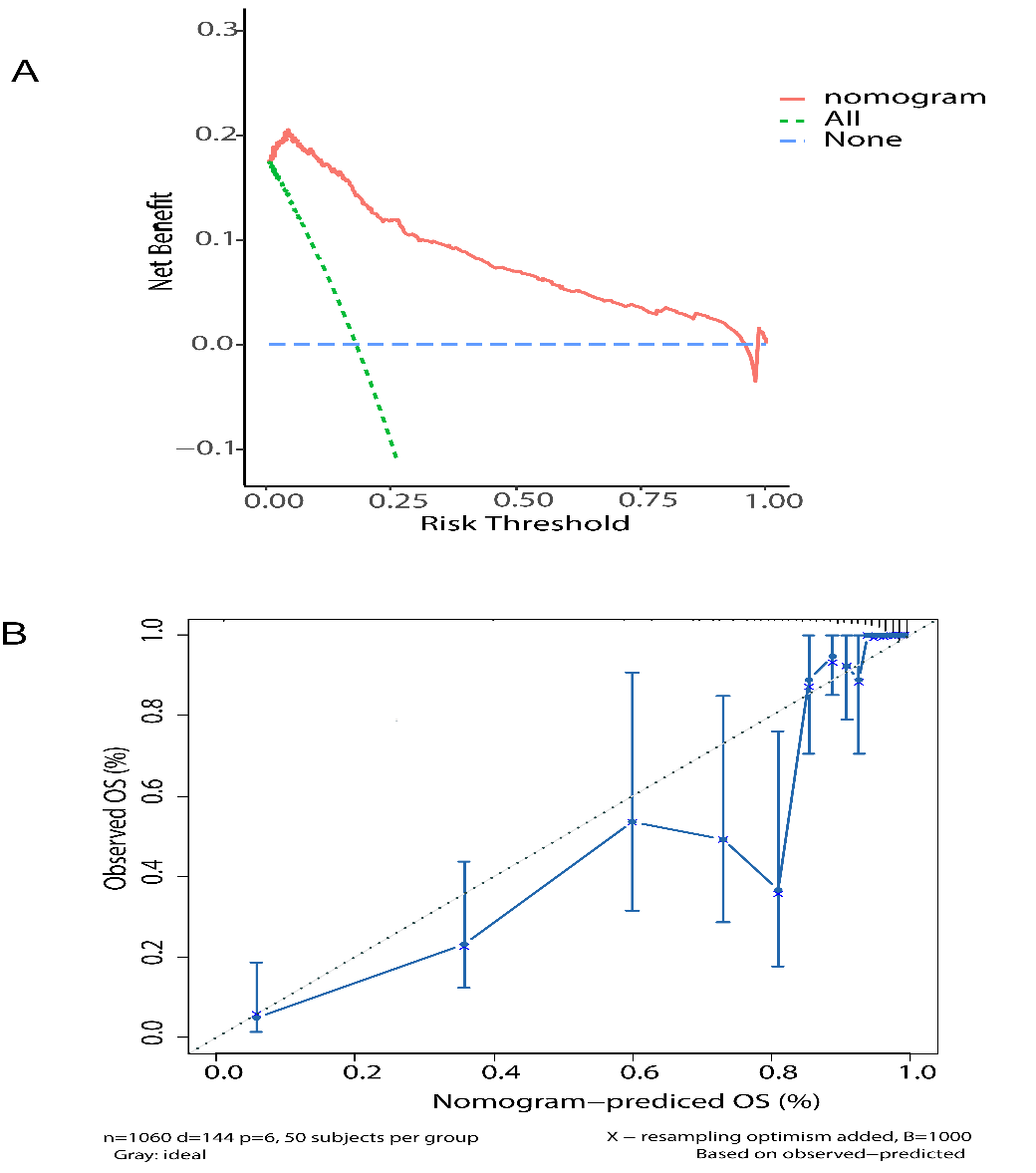


**Supplementary Figure S2.** **Comparison of the overall model including prognostic genes related to lipid metabolism and other models constructed in TCGA-BRCA data**

A: A clinical decision curve was plotted. According to the nomogram, a net benefit could be achieved when the probability of cervical lymph node metastasis ranged from 0.2 to 0.8, indicating excellent model performance B: The calibration graph was utilized to rectify the mean absolute error between the predicted values and the actual values.

## Supplementary FigureS3


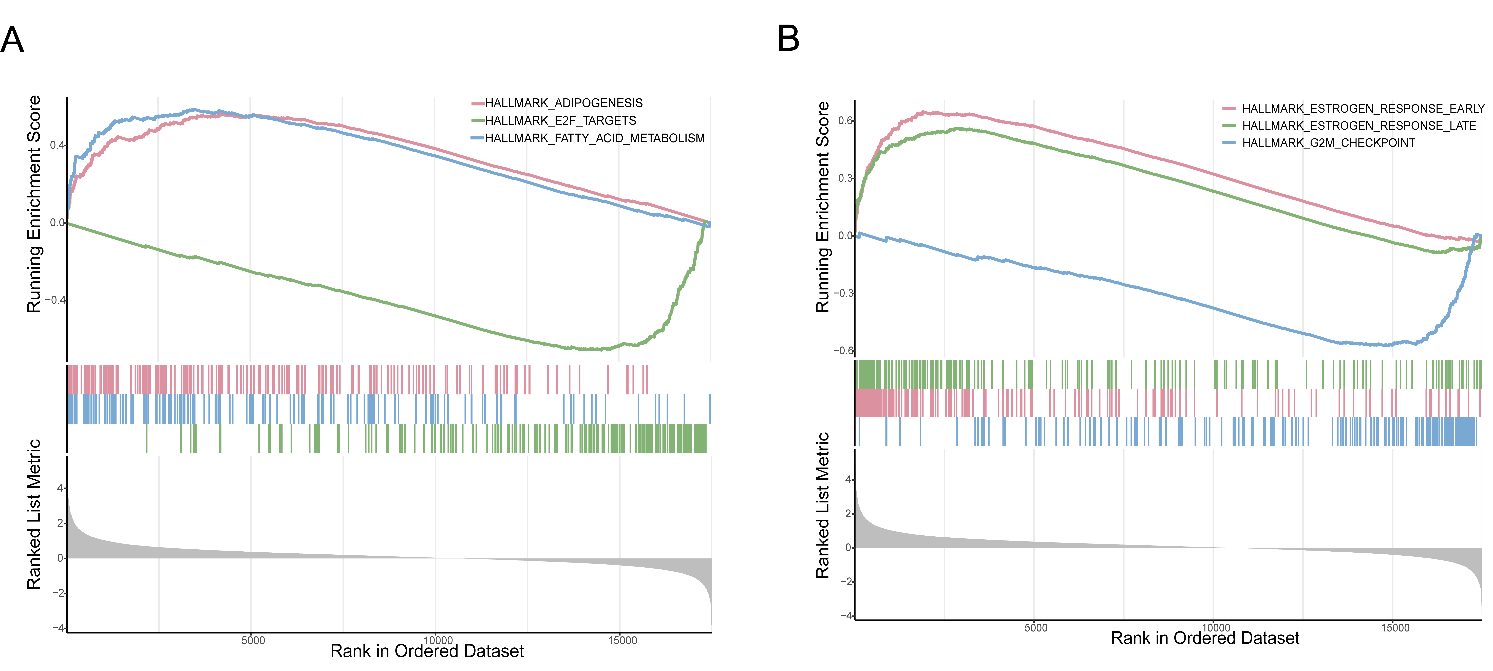


**Supplementary Figure S3.** **Clinical characteristics and pathway differences between high and low lipid metabolic-related prognostic gene score groups in TCGA data and their application in pan-cancer**

A: Adipogenesis, E2F targeting, and Fatty acid metabolism pathways were highly expressed in the high-score group; B: Early oestrogen response, Late oestrogen response, and the G2/M checkpoint were more enriched in the low-score group.

## Supplementary FigureS4


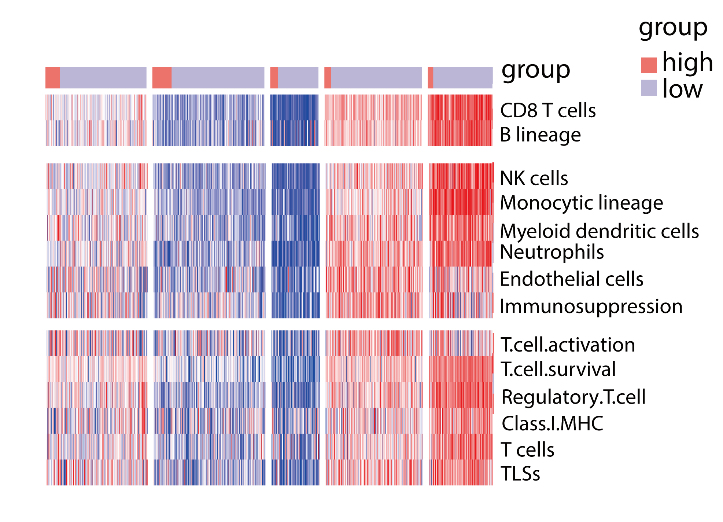


# Supplementary Figure S4. Differences in immune cell infiltration between high and low lipid metabolism prognostic score groups in TCGA-BRCA data

# Difference in abundance of 14 immune cells calculated by MCPcounter between high and low score groups of prognostic genes related to lipid metabolism in TCGA-BRCA data; However, we observed that the location of immune cells did not significantly differ among the two score groups for LMPGS 。

## Supplementary FigureS5


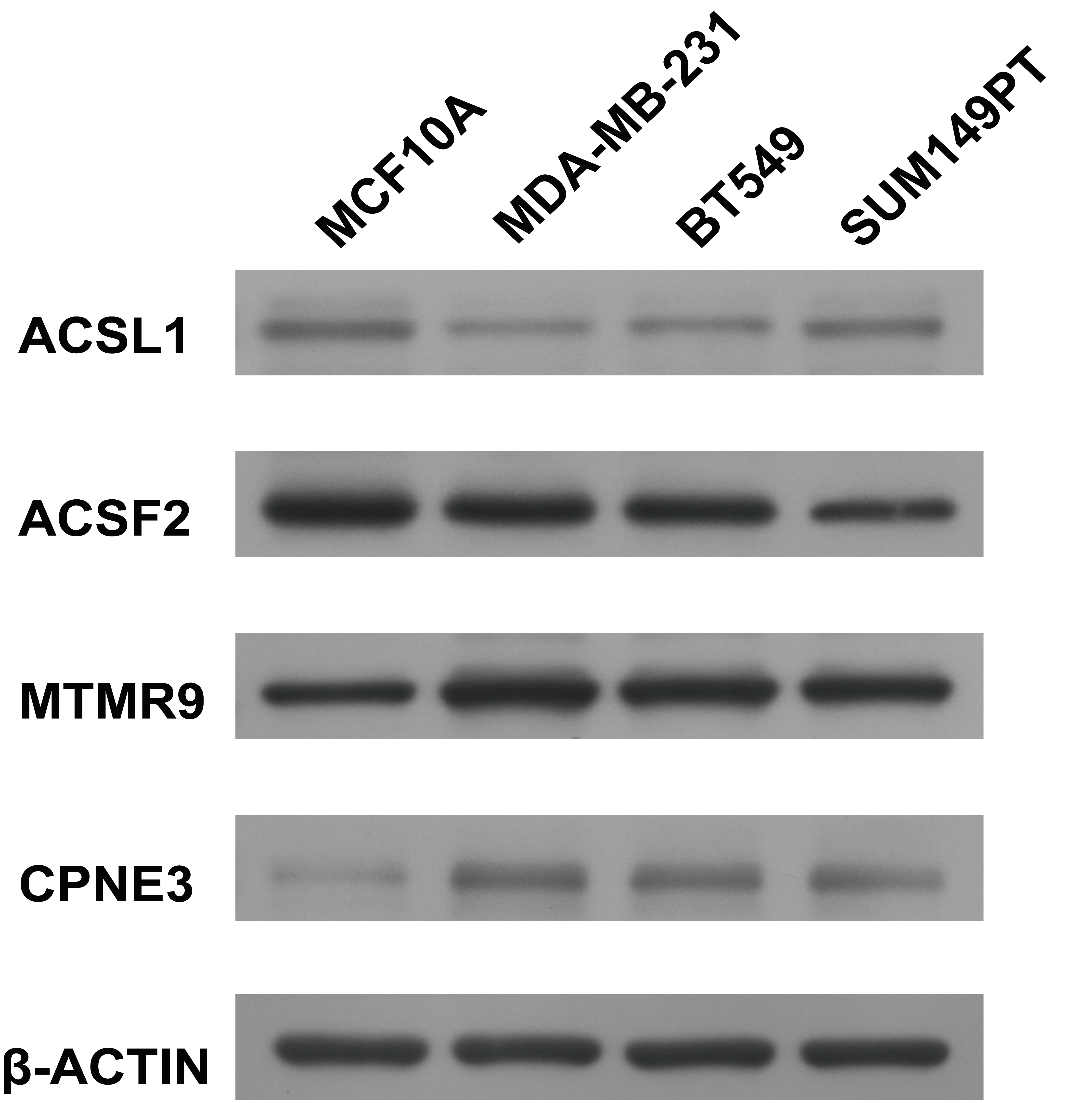


# Supplementary Figure S5. Verification of LMPGS hub genes expression.

MTMR9 and CPNE3 were highly expressed in MDA-MB-231 and BT549-SUM149PT breast cancer cell lines, and were low expressed in MCF10A non-tumorigenic breast cancer cell lines. ACSL1 and ACSF2 were lowly expressed in MDA-MB-231，BT549 and SUM149PT breast cancer cell lines, and were high expressed in MCF10A non-tumorigenic breast cancer cell lines.

## Supplementary FigureS6


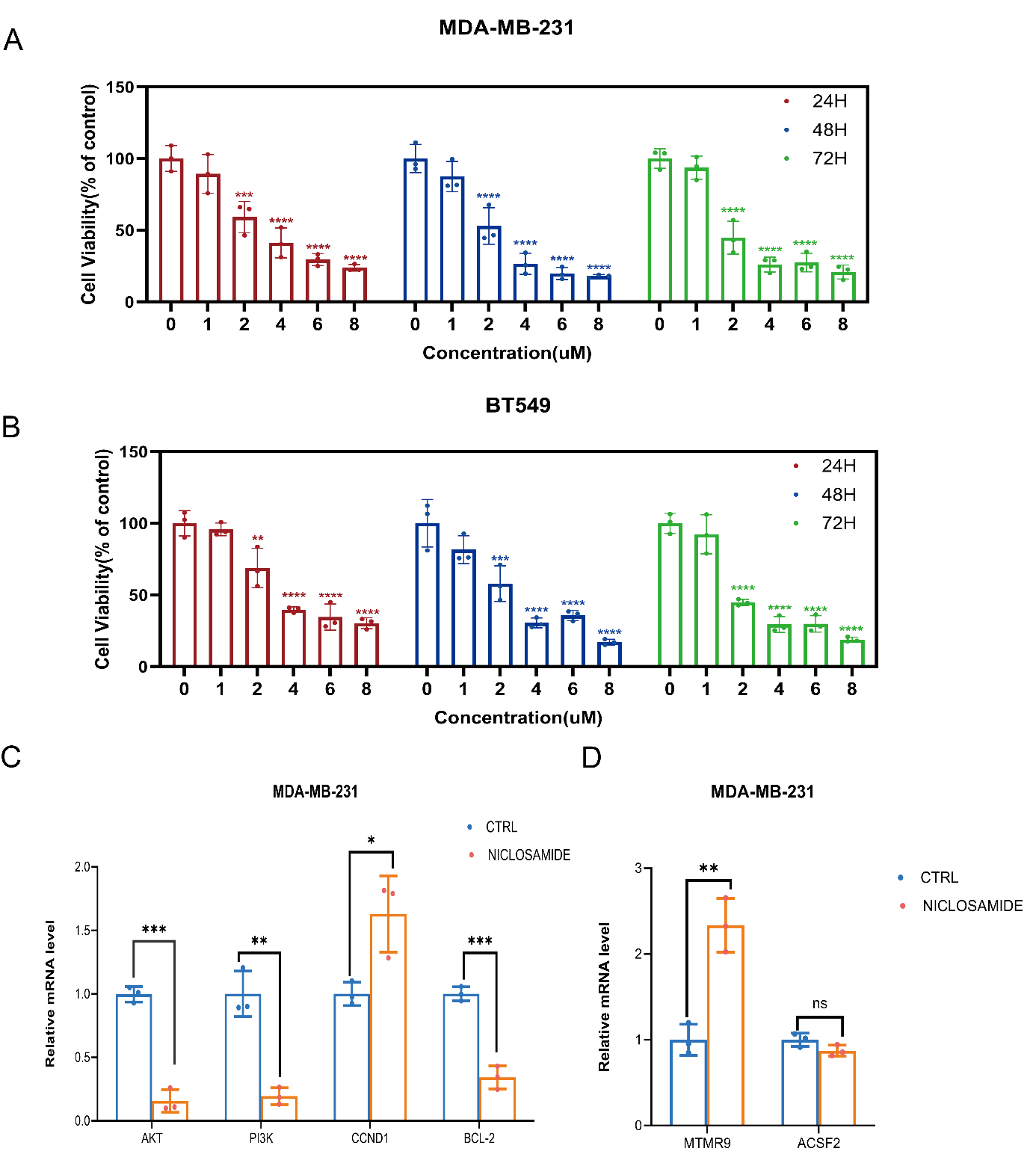


# Supplementary Figure S6. NICLOSAMIDE inhibits the growth of breast cancer cells and affects the expression of target genes

A,B: NICLOSAMIDE inhibits the growth of breast cancer cells. The cells were treated with different concentrations of drugs, and the cell survival rate was observed at 24H, 48H, 72H. NICLOSAMIDE at 2uM,4uM,6uM,8uM could significantly inhibit cell proliferation in both MDA-MB231 and BT549 cell lines. C: In MDA-MB-231, cells treated with NICLOSAMIDE (8uM) for 24h significantly increased the expression of proliferation-related genes AKT, PI3K and anti-apoptosis gene BCL-2. D: In MDA-MB-231, cells treated with NICLOSAMIDE (8uM) for 24h significantly increased the expression of MTMR9.No regulation of ACSF2 expression was observed. (ns stands for p value>0.05; * stands for 0.01< p value <0.05; ** stands for 0.001< p value <0.01; *** stands for p value <0.001).
